# Differential properties of NS1 glycoproteins in West Nile and Usutu viruses

**DOI:** 10.1080/22221751.2026.2667565

**Published:** 2026-04-29

**Authors:** Justine Revel, Jérémy Leroy, Stéphane Delbecq, Orianne Constant, Florent Henry Marty, Chaima Naili, Arthur Barthès, Anna Nagy, Jonas Schmidt-Chanasit, Dániel Cadar, Nor Yasmin Abd. Rahaman, Anne-Dominique Lajoix, Caroline Desmetz, Yannick Simonin

**Affiliations:** a Pathogenesis and Control of Chronic and Emerging Infections (PCCEI), INSERM, University of Montpellier, Montpellier, France; b Biocommunication in Cardio-Metabolism (BC2M), University of Montpellier, Montpellier, France; c Centre de Biologie Structurale (CBS), CNRS, INSERM, University of Montpellier, Montpellier, France; d National Reference Laboratory for Viral Zoonoses, National Public Health Center, Budapest, Hungary; e Department of Arbovirology and Entomology, Bernhard Nocht Institute for Tropical Medicine, Hamburg, Germany; f Faculty of Mathematics, Informatics and Natural Sciences, University of Hamburg, Hamburg, Germany; g Virus Metagenomics and Evolution Group, Bernhard Nocht Institute for Tropical Medicine, Hamburg, Germany; h Institute of Bioscience, Faculty of Veterinary Medicine, Universiti Putra Malaysia, Serdang, Malaysia

**Keywords:** West Nile virus, Usutu virus, NS1 glycoprotein, blood-brain barrier integrity, neuroinvasion

## Abstract

West Nile virus (WNV) and Usutu virus (USUV) are neurotropic orthoflaviviruses of the *Flaviviridae* family, transmitted primarily by *Culex* mosquitoes and maintained in enzootic cycles involving birds. While WNV is a well-established human pathogen causing hundreds of neuroinvasive cases annually in Europe, USUV has emerged more recently, with fewer documented human infections but increasing evidence of neurovirulence. The viral nonstructural protein 1 (NS1) plays a central role in orthoflavivirus pathogenesis by modulating host immune responses, disrupting endothelial barrier integrity, and facilitating viral dissemination. However, the functional and biochemical properties of NS1 from WNV and USUV remain poorly characterized. We combined *in vitro*, *in vivo*, and clinical approaches to compare NS1 secretion, stability, and its impact on blood-brain barrier. Our results show that WNV NS1 is secreted at significantly higher levels, exhibits greater thermal stability, and disrupts brain endothelial barrier integrity *in vitro*. In contrast, USUV NS1 is secreted less efficiently, is slightly less stable, and does not compromise blood-brain barrier integrity, despite inducing distinct transcriptional responses in brain endothelial cells. In mice, WNV infection led to higher serum NS1 levels and stronger systemic inflammation than USUV. Clinically, WNV NS1 was detected mainly in patients with neurological symptoms, whereas USUV NS1 remained undetectable in all cases. Altogether, these findings reveal differential NS1 properties between these closely related viruses, with key implications for orthoflavivirus diagnosis and neurovirulence mechanisms.

## Introduction

WNV and USUV, both originating from Africa, are closely related viruses in the genus *Orthoflavivirus* within the family *Flaviviridae*. They are maintained through an enzootic cycle, mainly involving birds as amplifying hosts and Culex mosquitoes as the primary vectors [1,2]. They have gained increasing public health attention due to their recent emergence and ongoing circulation in Europe, especially in the southern and eastern parts of the continent [3,4]. Although human infections often go unnoticed, both viruses can occasionally lead to severe neurological disease, particularly WNV [5,6].

WNV is well known for causing neuroinvasive disease in humans and horses, including encephalitis, meningitis, and acute flaccid paralysis, which can sometimes be accompanied by long-term neurological sequelae such as cognitive impairment, motor deficits, and sensory dysfunction [7]. WNV is genetically classified into multiple lineages, with up to nine described to date [8]. Severe outcomes are mainly linked to lineages 1 and 2, which have caused recurrent outbreaks in Central and Southern Europe since the early 2000s, with increased viral circulation in recent years. First identified in Eastern Europe in the early 2000s, WNV-lineage 2 has progressively expanded westward, becoming dominant in many regions since 2010, contributed to the rising number of neuroinvasive cases reported in Europe [9–11].

USUV has more recently emerged in Europe, however both viruses are now co-circulating in several regions. Although it is less neurotropic in humans than WNV, whose pathogenesis is far better studied and understood, accumulating evidence over the past decade suggests that its clinical impact has likely been underestimated. USUV has been implicated in several cases of neurological disease across Europe, including meningitis, encephalitis, and meningoencephalitis [6]. Serological and virological data suggest that human infections with USUV may be more widespread than currently recognized. This may be due to cross-reactivity with other co-circulating orthoflaviviruses, especially WNV, which can obscure accurate detection of USUV in certain regions, as well as the lack of systematic surveillance specifically targeting USUV [2,12]. Confirmed neuroinvasive cases have now been reported in several European countries highlighting USUV’s emerging role as a human neuropathogen [6,13,14]. USUV is divided into eight distinct genetic lineages, grouped into African (Africa 1, 2, 3) and European (Europe 1, 2, 3, 4, 5) clusters [15,16]. In Europe, human neuroinvasive cases have been linked to multiple USUV lineages; however, most cases are associated with the EU2 lineage, which is actively circulating and appears to exhibit higher virulence than other lineages [17,18].

WNV can markedly disrupt the human blood–brain barrier (BBB), causing endothelial dysfunction, increased permeability, and pronounced neurovascular inflammation, which leads to extensive immune-cell recruitment and severe neurological injury [19]. In contrast, USUV, while also neuroinvasive, does not appear to alter BBB integrity [19,20]. Thus, both viruses can infect and cross the human BBB, but with distinct outcomes. WNV infection of BBB cells results in significant endothelial dysfunction, pronounced neuroinflammation, and immune cell recruitment, consistent with previous studies. In contrast, although USUV can infect BBB cells and replicate at higher levels than WNV, it does not markedly compromise endothelial integrity [19].

Like other orthoflaviviruses, both viruses are icosahedral-enveloped viruses with a single-stranded positive-sense RNA encoding a polyprotein. This polyprotein is post translationally processed by cellular and viral proteases into three structural proteins: capsid (C), premembrane/membrane (prM/M), and envelope (E), and seven nonstructural proteins (NS1, NS2A, NS2B, NS3, NS4A, NS4B, and NS5). The non-structural protein 1 (NS1) plays a central role in the pathogenicity of several orthoflaviviruses [21]. This multifunctional glycoprotein is secreted early during infection and participates in multiple pathogenic processes, modulating innate immunity, disrupting vascular integrity, and contributing to neuroinvasion [21]. A comparative analysis of NS1 proteins from five orthoflaviviruses (DENV, ZIKV, WNV, JEV, and YFV) demonstrated that NS1 induces tissue-specific endothelial hyperpermeability rather than a uniform vascular response [22]. This tissue specificity is not limited to surface binding but also involves downstream steps such as NS1 internalization, which appears to be a key determinant of endothelial dysfunction.

The role of NS1 in WNV infections remains poorly defined. Current evidence suggests that NS1 contributes to WNV neurovirulence, primarily through its effects on cerebral endothelial cells and the associated inflammatory responses, whereas the role of NS1 in USUV has not yet been investigated. NS1 from orthoflaviviruses can be secreted as an extracellular hexamer (sNS1) and bind to endothelial cell membranes, leading to destabilization of intercellular junctions and the endothelial glycocalyx. This, in turn, increases vascular permeability and promotes leakage of fluids and proteins into surrounding tissues [21,23–27]. Simultaneously, NS1 can stimulate endothelial and immune cells to produce pro-inflammatory cytokines and vasoactive molecules, further amplifying vascular barrier disruption and creating a microenvironment that facilitates neuroinvasion [28,29]. Moreover, NS1 can be transported via extracellular vesicles, such as exosomes and microvesicles, allowing it to act at sites distant from the producing cells and extend its cytopathic effects on cerebral endothelium, thereby contributing to the spread of vascular leakage [30]. Thus, NS1 emerges as a key player in the cerebral pathogenesis of neuroinvasive orthoflaviviruses, particularly through its effects on endothelial permeability and neuroinflammation, and represents a relevant target for the development of diagnostic and therapeutic strategies.

As WNV and USUV are phylogenetically closely related viruses but are associated with distinct neurological outcomes, we compared the secretion and functional effects of NS1 from these two viruses using brain endothelial cells and *in vitro* BBB models. We also assessed NS1 prevalence in the serum of infected mice and in patients who were asymptomatic or presented with mild to severe forms of disease. Our results demonstrate that NS1 secretion and functional activity differ markedly between these closely related viruses, with important implications for understanding their pathogenicity and for the use of NS1 as a diagnostic marker of WNV infection.

## Materials and methods

### Ethics approval and consent to participate

Mice were bred and maintained in a biosafety level 3 (BSL-3) facility in accordance with the French Ministry of Agriculture and European institutional guidelines (Appendix A STE n°123). All experimental procedures were approved by the French ethics committee (approval n° 6773-201609161356607). For all patients, written informed consent was obtained in their respective countries of residence, in accordance with local regulations and with approval from the Institutional Review Board (IRB), specifically the National Public Health Center in Budapest.

### Patient cohort

A cohort of serum and cerebrospinal fluid (CSF) samples from patients infected with WNV or USUV was analyzed. WNV samples were obtained from Hungarian patients, while USUV samples originated from patients in Hungary and Germany (Supplementary Table 1). The cohort included 35.7% females (median age: 59 years; IQR: 47–71.5 years) and 64.3% males (median age: 66 years; IQR: 51–74.5 years). Patients presented either West Nile fever (WNF; median age: 49 years; IQR: 30–61 years) or West Nile neuroinvasive disease (WNND; median age: 67 years). WNF was mainly characterized by fever, exanthema, and headache, whereas WNND cases predominantly manifested with meningitis, encephalitis, meningoencephalitis, or encephalomyelitis, often accompanied by fever, headache, confusion, vomiting, dizziness, and episodes of unconsciousness. In all WNV-infected patients, the detected virus belonged to lineage 2 strains, whereas USUV infections involved the Europe 2 lineage. For WNV, CSF samples included 12 controls and 53 patients with neurological manifestations. Serum samples comprised 26 controls, 9 asymptomatic individuals, 23 febrile patients, and 62 patients with neurological manifestations. For USUV, serum samples included 25 controls, 12 asymptomatic individuals, and 4 patients with neurological manifestations. All infections and disease classifications were confirmed by RT-qPCR (using the cobas® WNV assay and USUV-specific RT-qPCR [31]) or IgM ELISA, ensuring accurate identification of WNV and USUV cases, as well as reliable differentiation between asymptomatic, febrile, and neurological manifestations. To address potential cross-reactivity with other endemic orthoflaviviruses, all cases were systematically tested for WNV, USUV and tick-borne encephalitis virus (TBEV) using serological assays (IgM, IgG, and IgA detection). WNV and TBEV serology was performed using Euroimmun’s CE-IVD-marked orthoflavivirus Mosaic indirect immunofluorescence assays (IFA), while USUV testing was carried out using in-house IFA methods. Additionally, IFA testing was combined with capture IgM ELISA (Focus Diagnostics), IgM and IgG ELISAs (Euroimmun), and IgG avidity ELISA (Euroimmun) tests to support diagnostic accuracy.

### NS1 production and characterization

#### Production and quality control

Synthetic genes encoding NS1 were obtained from IDT (Integrated DNA Technologies). Briefly, NS1 from the USUV Europe 2 strain (TE18982/Italy/2017; GenBank: MT784898.1) or from the WNV lineage 2 Greek strain (S36_GrM_2010/Thessaloniki; GenBank: MN481595.1) were synthesized with a C-terminal 6×His tag, flanked by EcoRV and NheI restriction sites. NS1 sequences were then cloned into the pFUSE-IgG1-Fc2 vector (Invivogen) via an EcoRV-NheI double digestion, resulting in NS1 expression under the control of the hEF1-HTLV promoter. The control vector corresponds to an empty pFUSE-IgG1-Fc2 plasmid, obtained during the cloning and which circularized without integrating the NS1 sequence, therefore lacking any insert. All constructs were verified by double or triple restriction digestion and sequencing.

Human embryonic kidney cells (HEK293 T; ATCC) were cultured at 37°C under 5% CO₂ in DMEM (Sigma) supplemented with 10% heat-inactivated FBS (Eurobio Scientific), 1% penicillin/streptomycin/L-glutamine (Sigma), and 1% G418 disulfate salt solution (Sigma). Eight million cells were plated in T150 flasks in complete medium. Twenty-four hours later, cells were transfected using polyethyleneimine (PEI; Polysciences, 1 mg/mL) at a ratio of 0.25 mg PEI per 30 µg plasmid DNA per T150 flask. The DNA/PEI complex was prepared in 150 mM NaCl, incubated for 20 min at room temperature, and added dropwise to the cells in serum- and G418-free DMEM. After 4 h, the medium was replaced with serum-free DMEM, and cells were incubated for 5 days.

Supernatants were collected and centrifuged (15 min, 3000 rpm, 4°C), diluted 1:1 with 2× phosphate buffer, and filtered through 0.22 µm membranes (Corning). NS1 protein was purified by affinity chromatography on an ÄKTA Pure system (Cytiva) using a 1 mL HisTrap Excel column (Cytiva) equilibrated with 20 mM disodium phosphate, 0.5 M NaCl, pH 7.4, and eluted with 500 mM imidazole. Fractions containing NS1 were concentrated and buffer-exchanged into DPBS (Sigma) using Amicon Ultra 30 kDa filters (Millipore). Purified proteins were sterilized by 0.22 µm filtration, aliquoted, and stored at −80°C. Protein concentration was determined by BCA assay (Pierce™ BCA Protein Assay Kit; Thermo Fisher Scientific) according to the manufacturer’s instructions, using bovine serum albumin as a standard. Protein integrity and purity were assessed by 10% SDS-PAGE followed by silver staining. Samples were prepared in Laemmli buffer, boiled for 5 min at 95°C, loaded alongside molecular weight markers (PageRuler™, Thermo Fisher Scientific), and separated at 150 V for 1 h. Gels were fixed in ethanol/acetic acid, sensitized with DTT, impregnated with silver nitrate, and developed until bands were visible. The preparations contained a mixture of NS1 oligomeric forms, where the major component seemed to be the dimer, as observed in Supplementary Figure 1.

#### MS/MS analysis of NS1

Gel electrophoresis was performed using a non-reducing loading buffer, followed by excision of the visible bands corresponding to NS1 and its various oligomeric forms (monomer migrating at ∼55 kDa, dimer, hexamer at ∼250 kDa; Supplementary Figure 1). In-gel digestion with trypsin was subsequently carried out. The resulting peptide samples were resuspended in 10 µL of buffer A (0.1% formic acid). An injection volume of 2–5 µL per sample was used for online analysis with a nano-flow HPLC system (RSLC U3000, Thermo Fisher Scientific) coupled to a mass spectrometer equipped with a nano-electrospray ionization source (Q Exactive HF, Thermo Fisher Scientific). Peptides were separated on a capillary column (0.075 mm × 500 mm, Acclaim PepMap 100, C18 reversed-phase, NanoViper, Thermo Fisher Scientific) using a 2–40% buffer B gradient over 128 min (buffer A: 0.1% formic acid; buffer B: 0.1% formic acid, 80% acetonitrile) at a flow rate of 300 nL/min. Spectral data were analyzed using MaxQuant v2.0.3.0 and Perseus v1.6.15.0, in combination with the leading FPP v3.5 script.

#### Differential scanning fluorimetry

The thermal stability of recombinant NS1 proteins was assessed using Differential Scanning Fluorimetry (DSF) on a Tycho NT.6 instrument (NanoTemper Technologies). Aliquots of NS1 proteins (control, WNV NS1, and USUV NS1) were thawed immediately before analysis. Each protein solution was loaded into standard-grade capillaries (NanoTemper) by inserting the capillary directly into the microtube, allowing passive capillary filling without pipetting. Filled capillaries were placed into the Tycho NT.6 sample holder, and measurements were performed according to the manufacturer’s default settings, monitoring intrinsic fluorescence at 330 and 350 nm during a controlled thermal ramp. Inflection temperatures (Ti) were automatically calculated using Tycho analysis software.

### Animals

#### Animal infections and sample collection

*Ifnar*^−^/^−^ mice (C57BL/6 background, adult, 8 weeks old) and Swiss mice (adult, 4 weeks old; young, 15 days old) were housed under BSL-3 conditions in compliance with institutional and European guidelines. Animals were inoculated subcutaneously with 10³ TCID₅₀ of West Nile virus (Lineage 2) or Usutu virus (Europe 2) in 50 µL PBS; control groups received PBS only. Group sizes were as follows: *Ifnar*^−^/^−^ mice (n = 15 controls, n = 18 USUV, n = 14 WNV), adult Swiss mice (n = 9 per condition), and young Swiss mice (n = 6 controls, n = 14 USUV). Animals were monitored daily for weight loss, neurological signs, and clinical deterioration; those reaching predefined humane endpoints were euthanized by cervical dislocation. Survival curves were generated for *Ifnar*^−^/^−^ and adult Swiss mice. Blood samples were collected at specific time points: day 3 post-infection for *Ifnar*^−^/^−^ mice infected with WNV; days 3 and 4 for *Ifnar*^−^/^−^ mice infected with USUV; days 3 and 6 for adult Swiss mice infected with WNV or USUV; and day 6 for young Swiss mice infected with USUV. Sera were obtained after centrifugation and stored at −80°C until analysis.

#### Intraperitoneal injection of NS1 recombinant proteins

Adult Swiss mice were injected intraperitoneally with 40 µg of purified recombinant NS1 proteins from WNV or USUV, or with CTL, 5 mice per condition. Animals were monitored daily for clinical signs and weight variation throughout the experiment. Blood samples were collected on day 4 for subsequent serum NS1 detection.

#### Tissue handling and RNA extraction

Injected mice were euthanized on day 4 and infected mice on day 6, or upon reaching predefined humane endpoints. Brains were carefully collected and processed according to the downstream application. A portion of the tissue was snap-frozen in liquid nitrogen and stored at –80 °C for virological or molecular analyses. Frozen tissues were kept at −20°C during dissection. For RNA extraction, approximately 30 mg of tissue was homogenized in RLT buffer supplemented with β-mercaptoethanol using a bead-based homogenizer. Homogenates were clarified by centrifugation, and total RNA was extracted using the RNeasy Mini Kit (Qiagen) according to the manufacturer’s instructions. RNA concentration and purity were assessed using a Nanodrop spectrophotometer, and samples were stored at –80°C until further use.

#### Histology and immunostaining

For histological analyses, the remaining brain tissue from injected mice was initially fixed in 4% paraformaldehyde (PFA) and, before paraffin embedding, refixed for 24 h in 10% neutral buffered formalin. Tissues were then embedded in paraffin, sectioned at 3 µm using a microtome at the RHEM facility (Biocampus, Montpellier), mounted on glass slides, and dried overnight at 37 °C. Immunohistochemistry was performed using established protocols. CD45 immunostaining was carried out with a rat anti-CD45 monoclonal antibody (clone 14-0451, Bioscience). Slides were scanned using a Hamamatsu NanoZoomer 2.0-HT slide scanner, and quantification of CD45-positive cells was performed with QuPath software.

### Viral strains

The following strains were used in this study: WNV lineage 1, WNV lineage 2, USUV Europe 2, USUV Europe 3, USUV Europe 5, USUV Africa 2, and USUV Africa 3. The origins of the viral strains were as follows: WNV lineage 1 (West Nile virus/Tunisia/2018); WNV lineage 2 (WNV-3125/France/2018) from ANSES, France; USUV Europe 2 (TE20421/Italy/2017) from Istituto Zooprofilattico Sperimentale, Emilia Romagna, Italy; USUV Europe 3 (USUV-HautRhin7315/France/2015), USUV Africa 2 (Rhône 2705/France/2015) and USUV Africa 3 (USUV-HauteVienne4997/France/2018) from ANSES, France; USUV Europe 5 (BNI507/2016/Germany) from Bernhard Nocht Institute for Tropical Medicine, Hamburg, Germany (Table 1). All viral strains were amplified no more than four times on Vero E6 cells before use. Viral stocks were prepared by infecting 70–80% confluent Vero E6 cells.

**Table 1.** Country, year of isolation and passage history of the USUV and WNV strains used in the study.

Lineage	Strains Name	Country of isolation	Year of isolation	Number of passages
Europe 2	TE20421/Italy/2017	Italy	2017	3
Europe 3	USUV-HautRhin7315	France	2015	4
Europe 5	BNI507/2016/Germany	Germany	2016	3
Africa 2	Rhône 2705	France	2015	3
Africa 3	USUV-HauteVienne4977	France	2018	4
WNV 1	West Nile virus/Tunisia/2018	Tunisia	2018	4
WNV 2	WNV-3125/France/2018	France	2018	3

### Cells

#### Infection of Vero E6 cells

Vero E6 cells (ATCC CRL1586) were maintained in Dulbecco’s Modified Eagle Medium (DMEM; Sigma) supplemented with 10% heat-inactivated fetal bovine serum (FBS; Dutscher), 1% penicillin-streptomycin solution (100X; Gibco), at 37°C under 5% CO₂. For experimental infections, sub-confluent cells were rinsed once with PBS, and viral inoculum at a multiplicity of infection (MOI) of 0.1 was added in a small volume of medium for 2 h at 37 °C with constant agitation. The inoculum was then removed, fresh medium was added, and cells were incubated for 6 days. At the end of the incubation period, culture supernatants were titrated and harvested, then stored at −80°C for subsequent analyses.

#### Viral titration by TCID₅₀ assay

Viral titers were determined by endpoint dilution assay on Vero E6 cells. Briefly, Vero E6 cells were seeded in 96-well plates at 1.5 × 10⁵ cells/mL and allowed to adhere for 3 h at 37°C with 5% CO₂. Viral supernatants were serially diluted (10-fold dilutions from 10^−^¹ to 10^−^⁹) in DMEM supplemented with 10% FBS. After removal of the culture medium, 100 µL of each dilution were added to six replicate wells per plate, and each sample was tested in duplicate. Plates were incubated at 37°C with 5% CO₂ for up to 7 days.

Cytopathic effects (CPE) were evaluated microscopically between day 5 and day 7 post-infection. Viral titers were calculated using the Reed–Muench method and expressed as TCID₅₀/mL.

#### Treatment of HBMEC with NS1

Primary human brain microvascular endothelial cells (HBMEC; CliniSciences) were thawed from cryovials (passage 5; ∼400,000 cells) and resuspended in EGM-2 complete medium (Lonza). Cells were cultured in T75 flasks pre-coated overnight at 37°C with 300 µL fibronectin (1 mg/mL; CliniSciences). Medium was changed every two days until confluence.

For treatments, fibronectin-coated 6-well plates were seeded with 80,000 cells/well in 2.5 mL EGM-2 medium. After 3 days at 37°C with 5% CO₂, cells were treated with USUV or WNV NS1 at 10 µg/mL or control in the same medium and refreshed daily by replacing half of the volume with NS1-containing medium for 4 days.

At designated time points (Day 0 and Day 4), supernatants were collected, clarified (5 min, 1000 g, 4°C), and stored at −80°C. Cells were washed twice with DPBS (Sigma) and lysed in 300 µL RLT buffer containing 1% β-mercaptoethanol for RNA extraction (RNeasy Mini Kit, QIAGEN), followed by DNase I treatment according to the manufacturer’s protocol.

### RT-qPCR

#### RT-qPCR in HBMEC

RNA was reverse-transcribed using LunaScript RT SuperMix (NEB), and qPCR analysis was performed for genes involved in endothelial permeability (hβ-actin, hOccludin, hZO-1) using the LC96 real-time PCR instrument (Roche).

#### PCR array in HBMEC

Using the same RNA samples, RT2 Profiler PCR Array Human Endothelial Cell Biology (PAHS 015ZA, 96-well format, Qiagen, 84 genes analyzed) was performed. Volumes of mix, cDNA, RNAse-free water, and cycling conditions were determined according to the manufacturer’s instructions. Genes without interpretable amplification curves were excluded from the analysis. Data were analyzed using Geneglobe (Qiagen), where the *p* values are calculated based on a student’s t-test of the replicate (2^(- Delta CT)) values for each gene in the control group and treatment groups, and *p*-values less than 0.05 are considered significant.

#### RT-qPCR in mice

Complementary DNA (cDNA) was synthesized from total RNA using the OmniScript RT Kit (Qiagen). Quantitative PCR was performed to assess the expression of genes associated with inflammation and endothelial function. In infected mice, *Il1b, Il6, Ccl5, Tnf, and Cxcl10* transcripts were quantified and normalized to a housekeeping gene (*Gapdh* or *Actb*). In subcutaneously injected mice, expression levels of *Il6, Il8, Tnf, Cxcl10, Ccl2, Ccl5, Il1b, Cflar, Ednra, Mmp2, Serpine1, Tgfb1, Timp1, Ocln, Cldn5, Tjp1, and Mmp9* were determined and normalized to *Actb*. Relative gene expression was calculated using the 2^−^ΔΔCt method.

### *In vitro* human-BBB model

#### Barrier model establishment

The human blood-brain barrier (BBB) model was established by co-culturing CD34^+^ umbilical cord blood-derived endothelial cells (hECs) with immortalized human brain pericytes for 6 days on 0.4 µm Transwell inserts (Costar) at 37°C and 5% CO₂ in MV2 endothelial growth medium (Promocell). Inserts were coated with Matrigel (0.2 mg/mL; Corning) on the apical side for hEC seeding and with 0.2% gelatin on the basal side for pericytes. This co-culture induced differentiation of hECs into brain-like endothelial cells (hBLECs), reproducing key features of human brain microvascular endothelial cells (HBMECs). Medium was refreshed every 2 days.

Barrier integrity was assessed using the Lucifer Yellow (LY) permeability assay (50 µM; Life Technologies). LY was prepared 1:70 in sterile-filtered Ringer’s solution (0.22 µm) containing: NaCl 8.8 g/L, KCl 387 mg/L, CaCl₂ 244 mg/L, MgCl₂·6H₂O 40.6 mg/L, NaHCO₃ 504 mg/L, HEPES 1.19 g/L, glucose 504 mg/L, pH 7.4. Inserts were incubated in wells containing 1.5 mL Ringer’s solution, and 300 µL LY was added to the apical chamber. After 20 min at 37°C, inserts were transferred to new wells with 1.5 mL Ringer’s solution for two additional 20-min incubations. Basolateral samples were collected, and fluorescence was measured in triplicate using a TECAN plate reader. Permeability coefficient (Pe) was calculated, with Pe ≤ 1 × 10^−^³ cm/min indicating an intact barrier, and Pe > 1 × 10^−^³ cm/min indicating disruption.

#### NS1 treatment in BBB model

For BBB models with Pe ≤ 1 × 10^−^³ cm/min, recombinant USUV or WNV NS1 or control was added at 10 µg/mL to the apical compartment in MV2 medium, renewed on days 2, 5, and 7. Barrier permeability was assessed on day 9 using the Lucifer Yellow assay as described above.

### ELISA assays

#### Cytokine multiple assays

A ProcartaPlex Mouse Cytokine and Chemokine Convenience Panel 1A 36-plex (Thermo Fisher Scientific) was used according to the manufacturer’s instructions to quantify inflammatory factors in mouse sera. Measurements were performed using a Luminex MAGPIX system (Thermo Fisher Scientific), and data were analyzed with GraphPad Prism 9.

For *Ifnar*^−^/^−^ mice, the following cytokines and chemokines were measured:

IL-1α, IL-1β, IL-3, IL-4, IL-6, IL-10, IL-12p70, IL-13, IL-15, IL-17A, IL-18, IL-28, IL-31, IFN-α, IFN-γ, GM-CSF, TNF-α, CCL2, CCL3, CCL7, CCL11, CXCL1, CXCL2, CXCL10, IL-5, CCL4, CCL5, IL-2, IL-9, IL-23, IL-27, LIF, IL-22, CXCL5, and M-CSF.

For Swiss mice, the following cytokines and chemokines were quantified:

IL-6, IL-22, IL-31, TNF-α, CCL2, CCL4, CCL7, CCL11, CXCL1, CXCL2, CXCL10, IL-1α, CXCL5, and IL-12p70.

#### Quantification of WNV or USUV NS1 protein

The concentration of NS1 protein was determined by sandwich ELISA. High-binding 96-well plates (MaxiSorp™, Nunc) were coated overnight at 4°C with 100 ng per well of the corresponding anti-NS1 monoclonal antibody (USUV: OAEF00644, CliniSciences; WNV: Ab253236, Abcam). Wells were then blocked for 1 h30 min at 37°C with PBS containing 2% BSA (Sigma) for USUV or 2% milk for WNV. Samples were diluted 1:5 in the respective blocking buffer (PBS-2% BSA for USUV; PBS-1% milk for WNV) and added at 100 µL per well, alongside standard curves of recombinant NS1 proteins (USUV: 200–0 ng/mL; WNV: 400–0 ng/mL). After 1 h30 min incubation at 37°C, wells were washed three times with PBS containing 0.1% Tween-20, followed by incubation for 1 h at 37°C with the primary anti-NS1 antibody (USUV: OAEF00645, CliniSciences), previously conjugated using the Flex kit (Proteintech) at 1:1300 dilution; WNV: PA5-111988 (Invitrogen) at a 1:2000 dilution. For WNV, after further washes, an additional 45 min incubation with a HRP-conjugated anti-rabbit secondary antibody (Ab6721, Abcam, 1:10,000) was carried out. After further washes, detection was performed with 100 µL TMB substrate solution (Promega), followed by stopping the reaction with 50 µL 3 M sulfuric acid. Absorbance was measured at 450 nm (TECAN microplate reader).

### Statistical analysis

All statistical analyses were performed using GraphPad Prism 9 (GraphPad Software). Data are presented as mean ± SEM. Differences were considered statistically significant at *p* ≤ 0.05. Data distribution was assessed and did not meet the criteria for normality; therefore, non-parametric statistical tests were applied. Comparisons among three or more groups were performed using the Kruskal–Wallis test, while comparisons between two groups were conducted using the Mann–Whitney test. Correlations were evaluated using Spearman’s rank correlation coefficient (r). Sample sizes, as well as the number of biological and/or technical replicates, are provided in the figure legends.

## Results

### Differential NS1 protein secretion following infection with multiple WNV and USUV lineages

**Figure 1.**
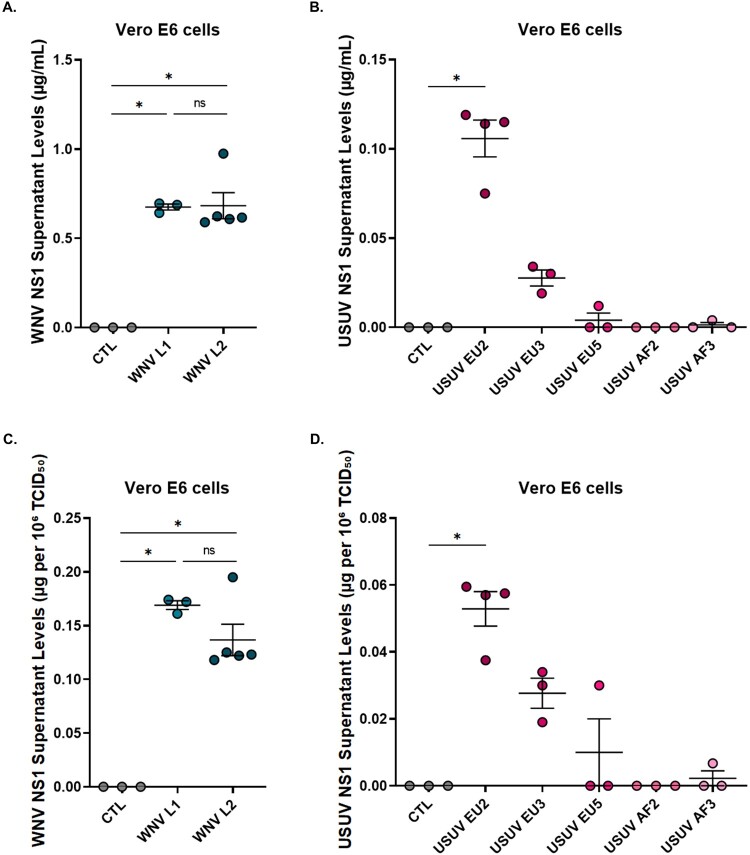
Quantification of NS1 protein concentrations in the supernatants of Vero E6 cells at 6 days post-infection with WNV and USUV. (A–B) Absolute NS1 concentrations measured by ELISA in supernatants of cells infected with (A) WNV or (B) USUV. (C–D) NS1 concentrations normalized to viral input (TCID₅₀/mL), allowing comparison of NS1 production per infectious unit for (C) WNV and (D) USUV. Data represent a single ELISA performed with 3 supernatants per condition, except for WNV L2 (n = 5) and USUV EU2 (n = 4). (*p ≤ 0.05).

First, NS1 protein secretion was assessed in cells infected with various lineages of WNV (L1 and L2) and USUV (EU2, EU3, EU5, AF2, and AF3). NS1 levels were quantified in the supernatants of infected Vero E6 cells using an in-house ELISA. WNV lineages exhibited comparable levels of NS1 secretion (Figure 1(A); WNV L1: 0.675 µg/mL; WNV L2: 0.682 µg/mL). In contrast, NS1 from USUV was predominantly detected in the supernatant of cells infected with the EU2 lineage, which was the only USUV strain showing statistically significant NS1 secretion (Figure 1(B)). Similar results were observed for USUV NS1 in endothelial cells (data not shown). Overall, the mean concentration of NS1 secreted following WNV infection (0.678 µg/mL) was approximately 6.4-fold higher than that observed for USUV EU2 (0.106 µg/mL). To account for potential differences in viral replication between strains, NS1 secretion levels were subsequently normalized to the corresponding infectious viral titers (TCID₅₀/mL) (Figure 1(C and D)). After normalization, no statistically significant difference in NS1 production per infectious unit was observed between WNV L1 and L2 (Figure 1(C)), with mean normalized values of 0.169 and 0.137 µg NS1 per 10⁶ TCID₅₀, respectively. Among USUV lineages, only the EU2 strain exhibited statistically significant NS1 production (0.053 µg per 10⁶ TCID₅₀) (Figure 1(D)). Importantly, WNV strains still exhibited approximately a 3-fold higher relative NS1 production efficiency compared to USUV lineages, confirming that the observed differences were not solely attributable to variations in viral input or replication efficiency.

Therefore, NS1 protein production is markedly higher in WNV lineages than in USUV lineages and varies substantially among USUV strains. While both WNV lineages exhibited robust NS1 secretion, detectable NS1 production among USUV isolates was largely restricted to the EU2 lineage. Based on these findings, WNV lineage 2 and the USUV EU2 lineage were selected for further analyses.

### Effect of NS1 from WNV and USUV on endothelial gene expression

**Figure 2.**
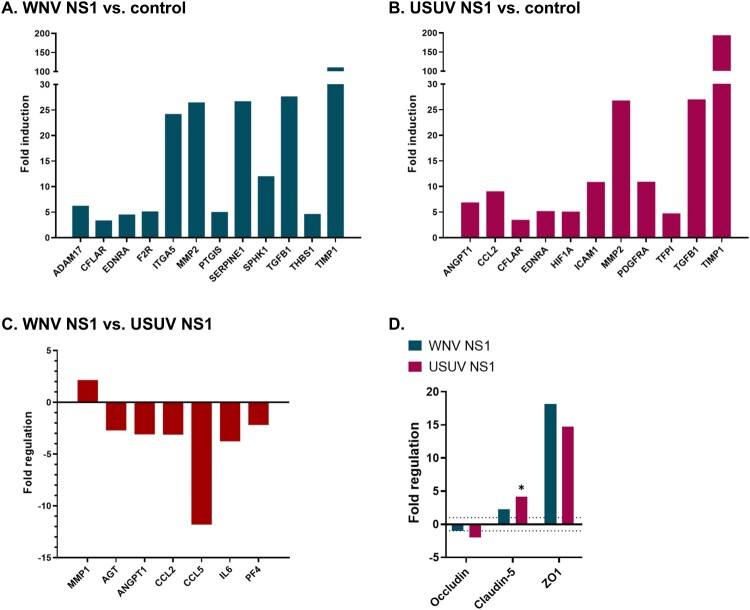
PCR array analyzing endothelial genes in HBMECs and showing significant expression differences. Cells were treated for 4 days with NS1 (10 µg/ml). (A) WNV NS1 vs. control, (B) USUV NS1 vs. control, and (C) WNV NS1 vs. USUV NS1. In panels A–C, all genes shown are statistically significant (*p* < 0.05), therefore no significance symbols are displayed. (D) Tight junction gene expression changes were detected in WNV or USUV NS1 conditions versus control; only one gene reached statistical significance and is indicated by an asterisk. Fold regulation of statistically significant genes normalized to control genes is indicated for 3 cell cultures per group. (Table S1).

To investigate the effects of NS1 from WNV and USUV on endothelial cell function, a key component of the BBB, we first produced recombinant WNV and USUV NS1 and verified its integrity by MS/MS analysis, with NS1 identified as the predominant protein in fractions corresponding to monomers, dimers, and oligomers (Supplementary Figure 1). Gene expression analysis was then performed using a PCR array (Human Endothelial Cell Biology) on primary human brain microvascular endothelial cells (HBMECs) treated with the recombinant NS1 proteins. Twelve genes were significantly upregulated following WNV NS1 treatment compared to control (Figure 2(A), Supplementary Table 2A), and 11 genes were upregulated following USUV NS1 treatment (Figure 2(B), Supplementary Table 2A). Several genes were commonly upregulated by both NS1 proteins, including *CFLAR*, *EDNRA*, *MMP2*, *TGFβ1*, and *TIMP1*, with *TIMP1* showing a particularly pronounced increase (fold regulation = 111.17 for WNV NS1 and 193.79 for USUV NS1). No significant downregulation was observed for any of the genes analyzed. Notably, significant differences were observed for certain genes between cells treated with WNV NS1 and those treated with USUV NS1 (Figure 2(C), Supplementary Table 2B). Specifically, *MMP1* expression was higher in WNV NS1-treated cells, whereas *AGT*, *ANGPT1*, *CCL2*, *CCL5*, *IL6*, and *PF4* showed lower expression in WNV NS1-treated cells compared to USUV NS1-treated cells. We then focused on genes encoding tight junction proteins, which are critical for BBB integrity (Figure 2(D)). Among these, *CLAUDIN-5* showed higher expression levels in USUV NS1-treated cells compared to controls. Marked differences in *ZO-1* expression were observed in cells treated with either USUV or WNV NS1; however, these differences did not reach statistical significance.

### Differential effects of WNV and USUV NS1 on a blood–brain barrier model

**Figure 3.**
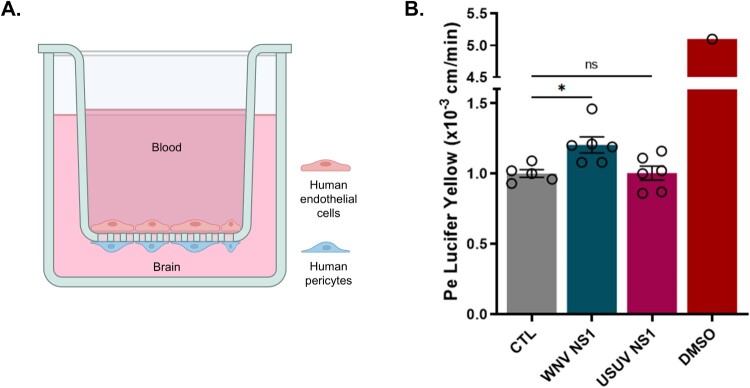
(A) The human in vitro BBB model used in this study consists of brain pericytes in the basolateral compartment, which promote the differentiation of CD34^+^-derived endothelial cells into human brain-like endothelial cells (hBLECs) on Transwell filters in the apical compartment. (B) Measurement of BBB permeability to Lucifer Yellow dye at day 9 post-treatment of cells with 10 µg/ml NS1. The experiment was performed twice, using 3 independent experiments per condition. (*p ≤ 0.05).

We then investigated whether NS1 proteins from WNV and USUV could affect BBB permeability and potentially contribute to the differences in neuroinvasion observed between these two viruses. To this end, we employed a human *in vitro* BBB model that recapitulates key features of the endothelial barrier. Human CD34^+^ cord blood-derived hematopoietic stem cells were cultured on inserts in co-culture with pericytes for 6 days to acquire BBB characteristics (Figure 3(A)). These human brain-like endothelial cells (hBLECs) are capable of forming tight junctions and express specific transporters, making them suitable for studying molecular and cellular passage across the BBB [32]. To further assess whether BBB integrity could be affected by WNV and USUV NS1, we measured Lucifer Yellow (LY) transport across the BBB model, a sensitive indicator of barrier permeability (Figure 3(B)). The permeability coefficient (Pe) in mock-treated controls was below 1, consistent with a “tight” BBB. Small but significant increases in Pe were observed following WNV NS1 treatment, indicative of a more permeable barrier, whereas USUV NS1 had no detectable effect (Figure 3(B)). The effect observed with WNV NS1 closely mirrored what was previously described after exposure to the whole virus [19]. These results suggest that WNV NS1 may contribute, at least in part, to the disruption of barrier integrity, whereas USUV NS1 appears not to affect BBB integrity *in vitro.*

### In vivo plasmatic and brain inflammatory responses following WNV and USUV infection in mice

We next investigated the potential effects of NS1 from WNV and USUV in mouse models, starting by examining the impact of infection on the cytokine profile in mouse serum. Immunocompetent Swiss adult mice were infected with both viruses. Because USUV poorly replicates in immunocompetent mice and typically induces only mild or no symptoms [33], mice lacking the type I interferon receptor (*Ifnar^−^/^−^*) were also used to study infections with USUV and WNV [19,34].

**Figure 4.**
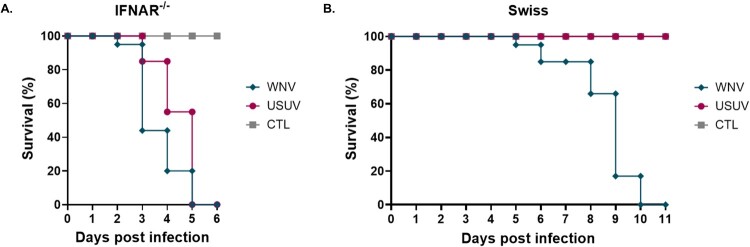
Survival curves of (A) *Ifnar*^−^/^−^ mice and (B) Swiss adult mice following infection with WNV or USUV.

In Ifnar^−^/^−^ mice, infection with either WNV or USUV resulted in rapid and complete mortality, with all animals succumbing within 5 days post-infection (Figure 4(A)), and similar viral titers detected in the blood for both viruses (1 × 10^6^ TCID₅₀/mL). In contrast, outcomes differed markedly in immunocompetent Swiss adult mice (Figure 4(B)). WNV infection caused progressive mortality starting at day 6 and reaching 100% by day 10 post-infection, whereas USUV-infected animals showed no mortality, with survival rates comparable to those of the control group. No viral replication was detected for USUV in the brains of immunocompetent adult mice, in contrast to WNV, for which a mean viral titre of 2 × 10^6^ TCID₅₀/mL was measured.

**Figure 5.**
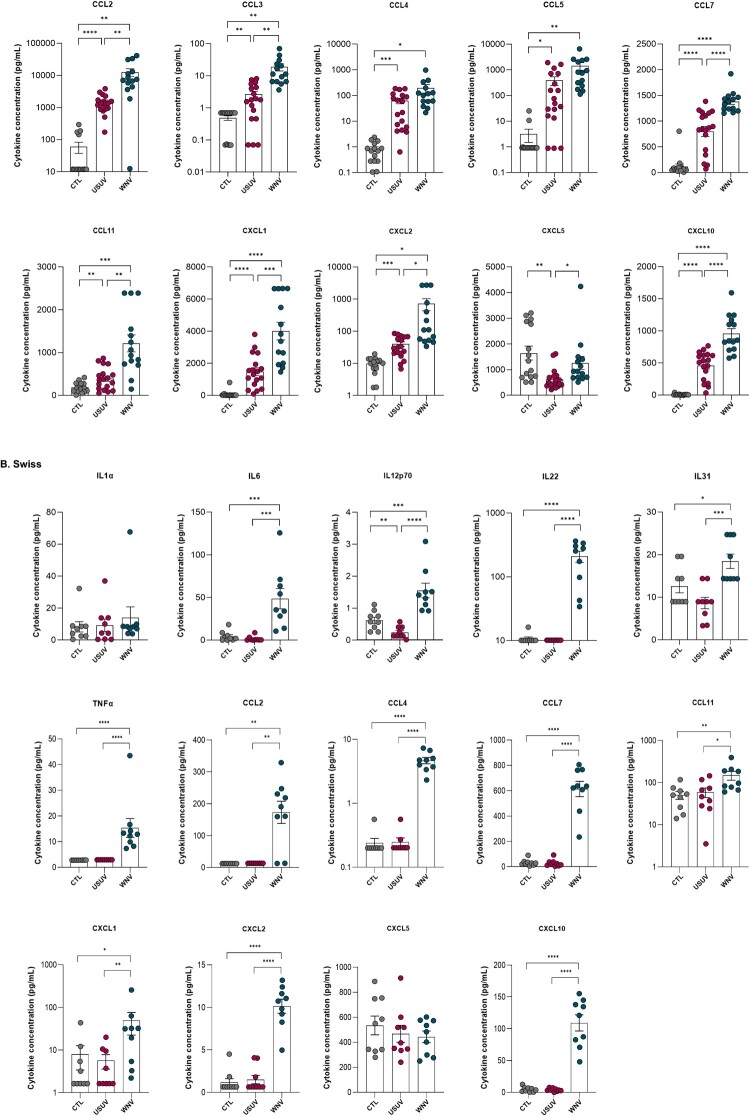
Continued.

We then characterized the cytokine expression profile in the sera of infected mice (Figure 5, Supplementary Table 3). In the immunocompromised *Ifnar*^−^/^−^ model (Figure 5(A), Supplementary Table 3A), infection with both viruses led to marked alterations in multiple inflammatory mediators compared with controls. These changes could be categorized into three distinct patterns. First, several mediators were significantly upregulated following infection with both USUV and WNV, with WNV inducing significantly higher levels than USUV (IL-1α, IL-1β, IL-3, IL-4, IL-6, IL-10, IL-12p70, IL-13, IL-15, IL-17A, IL-18, IL-28, IL-31, IFN-α, IFN-γ, GM-CSF, TNF-α, CCL2, CCL3, CCL7, CCL11, CXCL1, CXCL2, CXCL10). Second, some mediators were significantly elevated in response to both viruses compared with controls, without differences between WNV and USUV (IL-5, CCL4, CCL5). Third, a subset of mediators was specifically increased in WNV-infected mice only, compared with both controls and USUV-infected animals (IL-2, IL-9, IL-23, IL-27, LIF). Two mediators exhibited distinct patterns that deserved separate consideration. IL-22 was elevated in both WNV- and USUV-infected mice, although the increase reached statistical significance only in WNV-infected animals. In contrast, CXCL5 was specifically reduced following USUV infection compared with both controls and WNV-infected mice. Overall, these results demonstrate robust systemic activation of inflammatory mediators in response to viral infection, with a particularly pronounced response induced by WNV. While both viruses trigger a broad inflammatory response, they also appear to engage distinct inflammatory pathways.**Figure 5.**
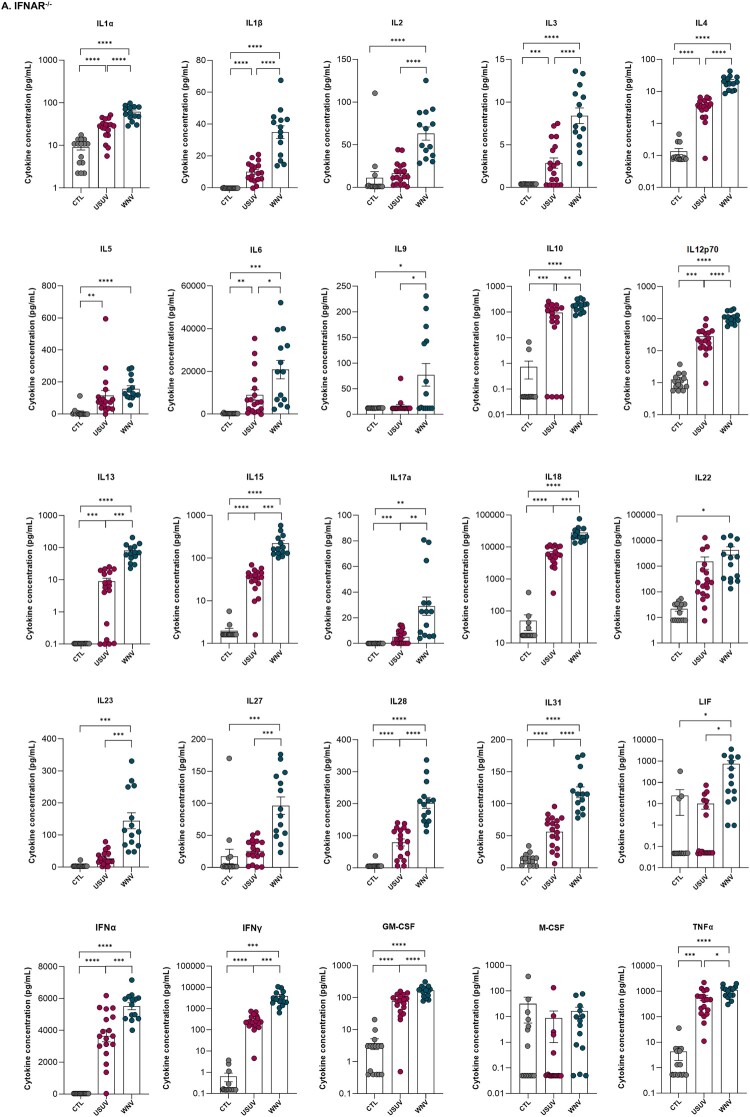
Quantification of serum protein concentrations in (A) *Ifnar*^−^/^−^ mice and (B) Swiss adult mice infected with WNV or USUV, measured by multiplexed ELISA at 3 days post infection. For *Ifnar*^−^/^−^ mice: n = 15 CTL, n = 18 USUV, and n = 14 WNV. For Swiss mice: n = 9 per condition. (*p ≤ 0.05; **p ≤ 0.01; ***p ≤ 0.001; ****p ≤ 0.0001).

In immunocompetent Swiss adult mice (Figure 5(B), Supplementary Table 3B), WNV infection significantly increased several pro-inflammatory cytokines, including IL-6, IL-22, IL-31, TNF-α, CCL2, CCL4, CCL7, CCL11, CXCL1, CXCL2, and CXCL10, compared with controls and USUV-infected mice. IL-12p70 was also upregulated following WNV infection but was downregulated in USUV-infected animals relative to controls. These findings further confirm that the systemic inflammatory response is markedly stronger during WNV infection than during USUV infection, particularly in an immunocompetent context.

We also assessed brain cytokine expression by RT-qPCR in immunocompetent Swiss adult mice, focusing on key mediators of neuroinflammation, including pro-inflammatory interleukins (*IL-1β, IL-6*), chemokines involved in immune cell recruitment (*CCL5, CXCL10*), and the major inflammatory cytokine *TNF-α*. No evidence of brain inflammation was observed in USUV-infected animals, as indicated by the lack of upregulation of these genes, in contrast to WNV-infected mice (Table 2). The absence of cytokine induction in USUV-infected mice is consistent with the lower susceptibility of immunocompetent mice to USUV compared with WNV.

**Table 2.** Expression levels of inflammatory genes in the brains of Swiss mice infected with USUV or WNV at 6 days post infection, evaluated by RT-qPCR.

Gene	WNV (mean ± SD)	USUV (mean ± SD)
IL-1β	6 ± 0.5	1.2 ± 0.1
IL-6	4.5 ± 0.4	1.1 ± 0.05
CCL5	7.9 ± 0.6	1.2 ± 0.08
TNF-α	12 ± 0.7	1.3 ± 0.1
CXCL10	62 ± 2.5	1.1 ± 0.07

Note: For each group, 6 mice were analyzed; fold regulation of statistically significant genes normalized to control genes is indicated.

Overall, WNV infection induced markedly stronger inflammatory responses than USUV infection, both in immunocompromised and immunocompetent models, highlighting the virus-specific differences in pathogenicity and the limited ability of USUV to trigger systemic or brain inflammation in immunocompetent hosts.

### Differential detection of NS1 in the serum of WNV- and USUV-infected mice

**Figure 6.**
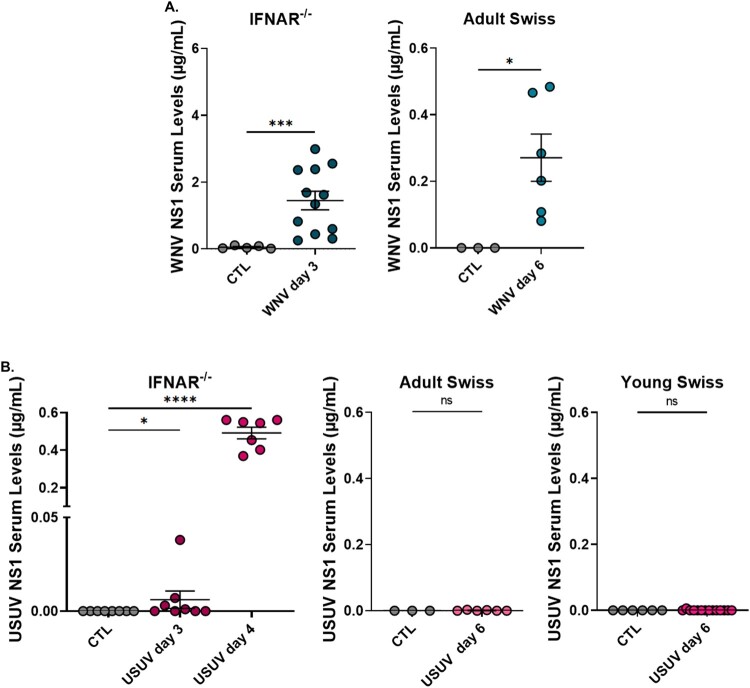
Quantification of NS1 protein concentrations in the serum of mice following subcutaneous injection of (A) WNV and (B) USUV, measured by ELISA. For WNV, *Ifnar*^−^/^−^ mice: n = 5 CTL, 12 WNV J3 and adult Swiss mice: n = 3 CTL, 6 WNV J6. For USUV, *Ifnar*^−^/^−^ mice: n = 8 CTL, 8 USUV J3, 7 USUV J4, young (suckling) Swiss mice: n = 6 CTL, 14 USUV J6 and adult Swiss mice: n = 3 CTL, 6 USUV J6. (*p ≤ 0.05; ***p ≤ 0.001); (*p ≤ 0.05; ****p ≤ 0.0001).

We next investigated whether the viral NS1 protein could be detected in the serum of mice infected with WNV or USUV. In the *Ifnar*^−^/^−^ model, WNV NS1 was detectable as early as day 3 post-infection, with a mean concentration of 1.446 µg/mL (Figure 6(A)). NS1 was also present in immunocompetent Swiss adult mice at day 6 post-infection, albeit at lower levels, with a mean concentration of 0.271 µg/mL, representing a 5.34-fold difference compared with *Ifnar*^−^/^−^ mice (Figure 6(A)).

Following USUV infection, NS1 was detectable only in *Ifnar*^−^/^−^ mice (Figure 6(B)). At day 3 post-infection, the mean concentration was 0.006 µg/mL, increasing to 0.492 µg/mL by day 4, corresponding to an 82-fold rise between the two time points. To investigate whether immune immaturity affects NS1 detection following USUV infection, both suckling and adult mice were analyzed. Suckling mice are described to be susceptible to USUV infection, similarly to adult *Ifnar^−^/^−^* mice, whereas immunocompetent adult mice are largely resistant. No NS1 was detected in Swiss suckling or adult mice at day 6 post-infection (Figure 6(B)). Consistent with these findings, viral replication in the brain was high in suckling mice, with a mean titre of 1 × 10⁷ TCID₅₀/mL, highlighting a clear contrast with immunocompetent adult mice. Direct comparison of NS1 serum levels between WNV and USUV infections revealed a striking difference. At day 3 post-infection, WNV-infected *Ifnar*^−^/^−^ mice exhibited a mean NS1 concentration 241-fold higher than that observed in USUV-infected *Ifnar*^−^/^−^ mice, despite similar viral titers in the blood (4 × 10⁶ TCID₅₀/mL). This pronounced disparity mirrors the *in vitro* results and underscores the markedly higher NS1 secretion associated with WNV infection.

### Effects of WNV and USUV NS1 in immunocompetent adult mice

**Figure 7.**
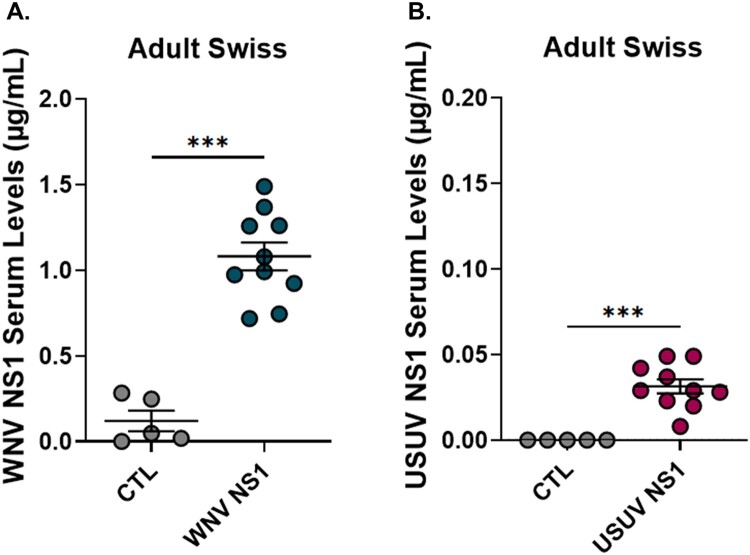
Quantification of NS1 protein concentrations in the serum of mice at day 4 following intraperitoneal injection of 40 µg of (A) WNV NS1 or (B) USUV NS1, measured by ELISA. For each group, 5 control mice and 10 NS1-injected mice were analyzed. (***p ≤ 0.001).

Following the differential detection of NS1 in infected mice, we next sought to investigate the *in vivo* effects of NS1 itself. Recombinant NS1 proteins from WNV and USUV were intraperitoneally injected into adult Swiss mice, and serum levels were measured (Figure 7). WNV NS1 was detected at an average concentration of 1.080 µg/mL, approximately 35-fold higher than USUV NS1, which was present at 0.031 µg/mL. This approach allowed us to directly compare the systemic exposure and potential biological effects of NS1 from the two viruses in an immunocompetent context.

**Figure 8.**
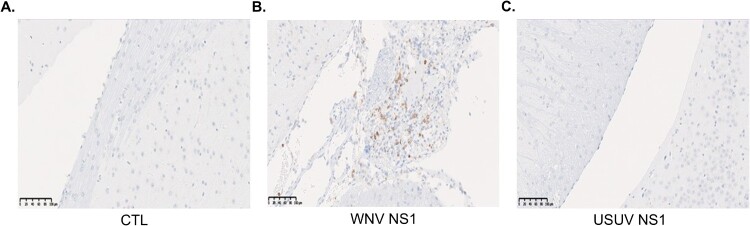
Histochemical analysis of brains from adult Swiss mice at day 4 post-intraperitoneal injection of (A) control, (B) WNV NS1, and (C) USUV NS1, using CD45 staining to assess T cell infiltration. Among the five mice per condition, focal recruitment only one WNV NS1-injected mouse exhibited focal recruitment of CD45^+^ inflammatory cells in the brain, whereas no such cellular recruitment was detected in the USUV NS1 or control groups.

We subsequently analyzed the presence of NS1 in the brains of injected mice. No NS1 from either WNV or USUV was detected. Consistently, no differences were observed between WNV and USUV regarding brain cytokines expression, including IL-6, IL-8, CCL2, CCL5, CXCL10, TNF-α, IL-1β, TGF-β1, MMP2, MMP9, occludin, claudin-5, ZO-1, CFLAR, EDNRA, serpine-1, and TIMP-1 (Supplementary Figure 2). Finally, CD45^+^ T cell infiltration was assessed by histochemical analysis (Figure 8). Among the five mice per condition, only one animal in the WNV NS1-injected group exhibited pronounced brain inflammation (Figure 8(B)), whereas no inflammation was observed in the USUV NS1-injected group (Figure 8(C)).

These results, within the limitations of the small number of animals, suggest that intraperitoneal injection of NS1 at the concentrations and time points studied does not induce widespread brain inflammation in mice, although focal inflammatory responses may occur in some individuals.

### Differential detection of NS1 in WNV and USUV patients

**Figure 9.**
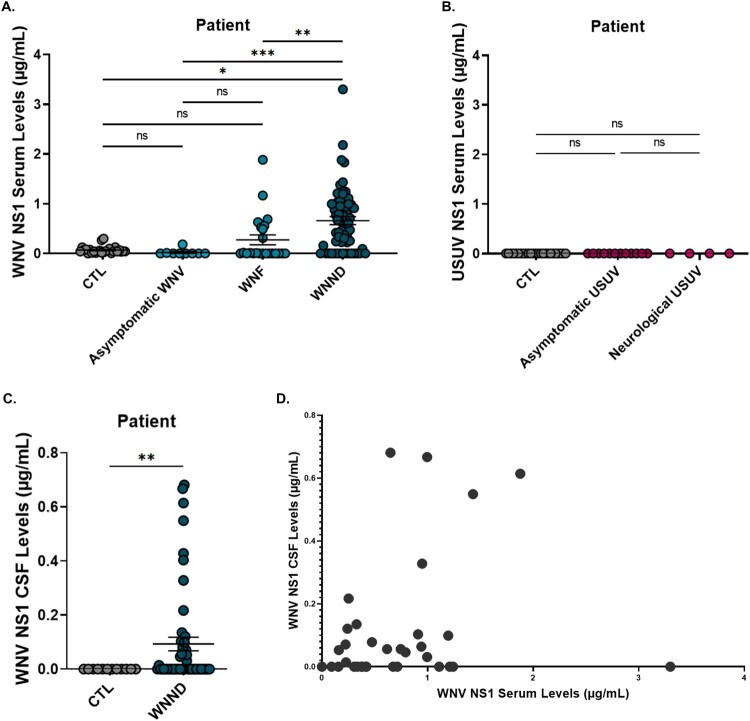
Quantification of NS1 protein in serum and cerebrospinal fluid (CSF) from infected patients, measured by ELISA. (A) NS1 levels in the serum of WNV-infected patients, including asymptomatic, febrile, and neurological cases. (B) NS1 levels in the serum of USUV-infected patients. (C) NS1 levels in the CSF of WNV-infected patients with neurological manifestations. (D) Correlation between NS1 protein levels in serum and CSF of WNV-infected patients (r = 0.506; 95% CI: 0.254–0.694; *p* < 0.001). (*p ≤ 0.05; **p ≤ 0.01; ***p ≤ 0.001).

We next assessed NS1 levels in patients infected with WNV or USUV. WNV NS1 was measured by ELISA in serum from control individuals, as well as in asymptomatic patients, patients displaying West Nile fever (WNF), or who developed West Nile neuroinvasive disease (WNND) following WNV infection. NS1 was only found in patients with WNND and was undetectable in asymptomatic cases (Figure 9(A)). Although some WNF patients showed low NS1 levels, these were not statistically significant. NS1 was also present in the cerebrospinal fluid (CSF) of patients with WNND (Figure 9(C)). Among these patients, serum and CSF NS1 levels did not differ significantly based on sex, age, or comorbidities (Supplementary Figure 3). A moderate, statistically significant positive correlation was observed between serum and CSF NS1 levels, with increases in serum NS1 often matching increases in CSF, and vice versa (Figure 9(D)). Conversely, USUV NS1 was undetectable in all patients, regardless of clinical presentation (Figure 9(B)).

Overall, NS1 was detectable exclusively in patients with neurological forms of WNV infection, whereas it remained undetectable in all USUV-infected patients regardless of clinical presentation. This striking difference highlights virus-specific expression patterns of NS1 and suggests that NS1 may serve as a potential biomarker for severe WNV infection, while its absence in USUV infections reflects either lower secretion levels or a different pathophysiological role of NS1 in this virus.

### Differential thermal stability of WNV and USUV NS1 proteins

**Figure 10.**
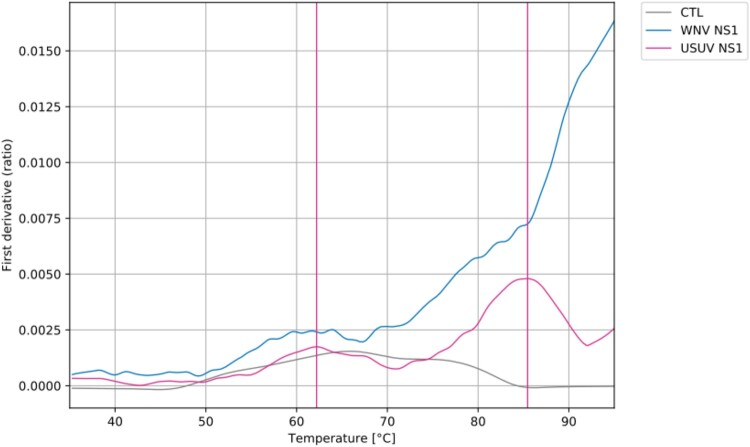
Differential Scanning Fluorimetry (DSF) analysis of CTL, WNV NS1, and USUV NS1. The first derivative of the fluorescence ratio (350 nm / 330 nm) reflects changes in the intrinsic fluorescence of aromatic amino acids, primarily tryptophans, enabling discrimination between the folded and denatured states of each protein. CTL does not exhibit a defined thermal transition and is shown for reference.

To investigate whether the differences observed between WNV and USUV NS1 in *in vitro* systems, mouse models, and patients could be at least partially attributed to intrinsic protein properties, we analyzed recombinant NS1 from both viruses. Thermal denaturation revealed clear differences in stability: WNV NS1 exhibited high thermal stability, with a gradual and continuous loss of structure occurring only at elevated temperatures. In contrast, USUV NS1 was slightly less stable, displaying a biphasic denaturation profile, with a minor unfolding event around 62°C followed by a more pronounced transition near 86°C (Figure 10). These intrinsic differences are consistent with the distinct secretion profiles and functional effects observed *in vitro,* suggesting that both protein stability and cellular behaviour contribute to the virus-specific properties of NS1.

## Discussion

To date, NS1 secretion has been extensively characterized for several orthoflaviviruses, including dengue virus (DENV), Zika virus (ZIKV), Japanese encephalitis virus (JEV) [35–37]. For these viruses, circulating NS1 has been proposed as a biomarker of neuroinvasion and as a potential therapeutic and vaccine target [28,38,39]. Moreover, NS1 is known to modulate endothelial permeability through mechanisms involving glycocalyx degradation, induction of metalloproteases, and junctional protein disruption [23,26,40]. In contrast to ZIKV and DENV, relatively little is known about the biological activity of WNV NS1 whereas USUV NS1 had remained completely unexplored. Previous studies have shown that WNV-infected cells secrete NS1 in substantial amounts, and that circulating NS1 can be detected in infected animal models [41–43]. NS1 administration prior to infection induces more severe clinical disease in WNV-infected red-legged partridges [44]. More recently, WNV NS1 has been shown to selectively alter brain endothelial cells and to promote blood–brain barrier permeability in both *in vitro* and *in vivo* models, supporting its role in viral neuroinvasion [22]. However, prior to this study, the presence of circulating NS1 in patients infected with WNV or USUV had not been investigated, and no comparative analyses between the two viruses were available.

We found that both WNV lineages 1 and 2 secreted comparable amounts of NS1 into the extracellular medium, whereas among USUV strains, only the Europe 2 lineage showed detectable secretion. On average, NS1 concentrations produced by WNV-infected cells were approximately six times higher than those measured following USUV infection. WNV and USUV NS1 proteins share 74.43% sequence identity, suggesting that relatively small differences in amino acid sequence can nonetheless result in substantial functional divergence, including differences in secretion efficiency and modulation of endothelial cells. These results indicate that NS1 secretion capacity varies not only between viral species but also among lineages. Whether these differences arise from intrinsic structural determinants of NS1 remains an open question. Structural data are available for WNV NS1, with crystallographic models deposited in the Protein Data Bank (PDB entries 4O6C and 4O6D), which reveal the conserved dimeric organization and secreted hexameric architecture characteristic of orthoflavivirus NS1 proteins. To date, no experimental structure has been reported for USUV NS1.

Given the ∼74% sequence identity between WNV and USUV NS1, homology modelling would be expected to predict a highly similar global fold, with predominantly localized surface variations. Accordingly, major structural rearrangements are unlikely to account for the functional differences observed here. Rather, subtle amino acid substitutions may influence oligomer stability, secretion efficiency, conformational dynamics, or interactions with host factors involved in endothelial modulation and inflammation. Importantly, currently available structural models represent static conformations and do not capture dynamic properties or interaction kinetics that may underline the systemic phenotypes described in this study. Further dedicated structural and biophysical investigations will therefore be required to determine how specific substitutions may modulate NS1 function. As an initial step, we provide a comparative sequence alignment of WNV and USUV NS1 in Supplementary Figure 4 to highlight conserved and variable regions that may guide future mechanistic studies. Thermal stability analyses further revealed that WNV NS1 is highly stable, whereas USUV NS1, although stable, exhibited slightly reduced stability and a biphasic denaturation profile with two distinct unfolding events. This two-step behaviour may reflect conformational rearrangements within the protein or an incomplete initial unfolding event leading to a second transition at higher temperatures. These findings may explain the low levels of NS1 detected in USUV-infected cells compared with WNV, as well as the observations in mouse models and patient sera. Indeed, while thermostable proteins may retain flexibility under physiological conditions (37°C), reduced thermal stability, as observed for USUV NS1, correlates with increased susceptibility to partial unfolding during intracellular trafficking, leading to recognition by ER quality control and ERAD-mediated degradation before secretion [45]. Thermostable proteins are generally more resistant to proteolysis and exhibit longer half-lives [46]. Structural determinants within the NS1 wing domain also influence secretion efficiency in orthoflaviviruses [47,48]. Once secreted, WNV NS1’s higher thermostability likely contributes to its prolonged half-life in serum and culture supernatants, resisting denaturation, aggregation, or extracellular proteolysis more effectively than USUV NS1. This dual characteristic, enhanced intracellular persistence leading to greater secretion, followed by greater extracellular persistence, could explain the observed differences in NS1 levels across our *in vitro*, *in vivo*, and patient samples. Importantly, this does not preclude functional flexibility, as WNV NS1 retains the conserved dynamic properties required for endothelial interactions and hexamerization. Extending DSF analyses to NS1 proteins from additional USUV and WNV lineages would be particularly informative to determine whether non-secreting strains share a common stability profile or whether lineage-specific mutations result in distinct unfolding behaviours.

For USUV, the absence of detectable NS1 secretion for most strains, except EU2, known to be the most virulent, suggests that this capacity may depend on the genetic sequence, with specific mutations that could participate in extracellular export. However, NS1 sequence comparison between the different USUV strains reveals only four amino acid differences overall (Supplementary Figure 5). It is therefore unlikely that these differences have a significant impact on the structure and function of NS1 in these different strains. It is also unlikely that this could explain the higher secretion levels of the EU2 strain. Observations in other orthoflaviviruses have identified factors that modulate NS1 flexibility, hexamer stability, and secretion capacity, supporting the hypothesis that determinants within the NS1 “wing” domain may influence its maturation, conformational dynamics, and extracellular export [36,37,49]. The distinctive secretion phenotype of NS1 from USUV EU2 may also result from factors beyond the intrinsic stability of the protein. Furthermore, previous studies have shown that the Europe 2 lineage replicates more efficiently than other USUV lineages, which could lead to earlier or increased detection of NS1 before significant denaturation occurs [17]. Disentangling the respective contributions of replication efficiency, NS1 stability, and secretion dynamics will require comprehensive analyses across multiple strains.

The PCR array analysis of human brain microvascular endothelial cells revealed that both NS1 proteins induce the expression of genes involved in tissue repair, wound healing, remodelling, and cell migration, as well as the maintenance of endothelial integrity and the regulation of inflammatory and coagulation responses, including CFLAR, EDNRA, MMP2, TGFβ1, and TIMP1. This aligns with previously described effects of WNV NS1 on endothelial activation and vascular remodelling [50,51] and suggests that NS1 coordinately modulates key mechanisms supporting endothelial protection and resilience, independently of the viral origin. Among these genes, TIMP1 (Tissue Inhibitor of Metalloproteinases-1) stands out with exceptionally strong overexpression, 111.17-fold for WNV NS1 and 193.79-fold for USUV NS1, far exceeding that of other upregulated genes (all below 30). This induction suggests an intense endothelial response aimed at counteracting metalloproteinase activation and preserving vascular barrier integrity in response to NS1-induced transcriptional stress. TIMP1 plays a central role in controlling extracellular matrix remodelling and stabilizing intercellular junctions. Its overexpression may also influence inflammation: beyond inhibiting MMPs, TIMP1 can activate signalling via CD63 and β1-integrins, promoting endothelial protection while also enhancing inflammation, for instance by facilitating leukocyte migration and cytokine production. Depending on the NS1 variant, this dual functionality could contribute to either a more protective response or, conversely, exacerbated inflammation. Moreover, a marked increase in ZO-1 expression was also observed in some endothelial cells treated with WNV and USUV NS1, although this difference was not statistically significant. This increase in ZO-1 mRNA may reflect a transcription–function uncoupling similar to that reported for DENV and ZIKV infections in endothelial cells. In these contexts, a compensatory upregulation of ZO-1 transcripts has been described, while the protein can undergo cytoplasmic relocalization or degradation (notably through MMP9/NS1-mediated mechanisms in ZIKV), ultimately being associated with increased permeability. Importantly, barrier function largely depend on proper cytoskeletal anchoring and junctional localization rather than on mRNA abundance [40,52]. Despite shared effects on some genes, others show distinct expression profiles depending on the NS1 protein. For example, WNV NS1 induces upregulation of MMP1, suggesting modulation of the extracellular matrix. In contrast, USUV NS1 increases CCL2, and upregulates claudin-5 and ANGPT1, both involved in junction maintenance and endothelial stability. These differences indicate that WNV and USUV NS1 activate distinct transcriptional pathways, with potential implications for regulating inflammation and endothelial integrity.

In our *in vitro* endothelial assays, we used a working concentration of 10 µg/ml of recombinant NS1. We acknowledge that this nominal dose exceeds the average NS1 levels measured in infected cell culture supernatants and in mouse serum, particularly in immunocompetent animals. However, these measurements reflect bulk or circulating concentrations and do not necessarily capture local NS1 accumulation in the immediate vicinity of highly infected foci. Our static 2D culture system is designed to model a local high exposure scenario, in which endothelial cells are uniformly and continuously exposed to NS1 in the absence of blood flow, clearance mechanisms, or spatial gradients. Notably, similar concentrations of orthoflaviviral NS1 (typically 5–10 µg/ml) are widely used to investigate endothelial dysfunction *in vitro* [25,53,54].

Functional analysis using an *in vitro* BBB model reinforces the differences observed between NS1 from WNV and USUV. Treatment with WNV NS1 results in a moderate but significant increase in barrier permeability, comparable to that observed following exposure to infectious virus and consistent with the activation of genes involved in matrix remodelling and inflammatory responses. In contrast, USUV NS1 does not induce significant changes in barrier-associated markers, despite a strong induction of TIMP1 and pro-inflammatory mediators such as CCL2, as previously described in endothelial cells treated with USUV NS1. These results indicate that NS1 contributes, at least in part, to WNV’s ability to disrupt BBB integrity, a key mechanism underlying neuroinvasion. Conversely, USUV NS1 appears to lack this activity, which may partly account for its limited neurotropism in humans. Further studies will be required to formally establish the contribution of NS1 to the differential neurovirulence of these two viruses.

In type I interferon receptor–deficient mice (Ifnar^−^/^−^), both infections cause rapid mortality, but systemic inflammatory responses are significantly stronger following WNV infection. Numerous pro-inflammatory mediators (such as IL-1, IL-6, TNFα, IFNγ, CCL2, and CXCL10) are expressed at substantially higher levels than in USUV-infected mice, reflecting exacerbated immune activation. In immunocompetent mice, only WNV infection induces mortality and a strong cytokine response, whereas USUV remains non-lethal and only weakly inflammatory. These differences likely reflect WNV’s greater ability to cross the blood–brain barrier and trigger severe neurovascular inflammation. In *Ifnar^−^/^−^* mice, serum NS1 concentrations are approximately 240-fold higher following WNV infection than after USUV infection. Moreover, in immunocompetent animals, only WNV NS1 is detectable, whereas USUV NS1 remains undetectable. Even after injection of recombinant proteins, WNV NS1 recovers at much higher concentrations than USUV NS1, suggesting greater stability or slower clearance. Histologically, WNV NS1 induces discrete foci of localized brain inflammation, characterized by CD45^+^ cell infiltration; these are absent following USUV NS1 injection and are not observed in all mice. These findings support the notion that WNV NS1 alone can trigger localized cerebral inflammatory responses, potentially contributing to the neurovascular damage observed during infection, with notable interindividual variability. Nevertheless, NS1 is not detected in the brains of treated mice, and NS1 alone is not sufficient to induce overt neurological symptoms, in contrast to what is observed following infection with infectious virus. A larger animal cohort, together with kinetic analyses using different doses of NS1, would be required to investigate more precisely the role of NS1 in murine models. This represents an important limitation of the present study, particularly due to the requirement for large amounts of recombinant NS1 to treat a substantial number of animals.

Our results show that WNV NS1 is predominantly detected in patients with neurological symptoms, including in the cerebrospinal fluid, highlighting its potential as a marker of neuroinvasion. In contrast, USUV NS1 was never detected, regardless of clinical presentation, consistent with its low secretion observed both *in vitro* and *in vivo*. These findings suggest that NS1 detection in serum or CSF could serve as a specific diagnostic indicator for neuroinvasive WNV infection. Moreover, the presence of NS1 in the CSF of patients with neurological symptoms following WNV infection reinforces the notion that WNV NS1 may cross the BBB and exert deleterious effects, unlike USUV NS1.

Taken together, while our findings highlight marked differences in NS1 secretion, stability, and endothelial effects between WNV and USUV, these intrinsic properties alone are unlikely to fully account for the differences in pathogenicity observed between the two viruses. Rather, disease outcome is influenced by complex host–virus interactions that can vary substantially across species and tissues. Notably, in certain avian hosts, USUV can display pathogenicity comparable to or even exceeding that of WNV, underscoring that additional viral and host determinants beyond NS1 properties contribute to virulence [55,56]. Factors such as viral replication dynamics, host immune responses, and tissue tropism are therefore also likely to play important roles in shaping clinical outcomes.

## Conclusion

Altogether, our results reveal that, despite the close genomic and structural similarities between WNV and USUV, their NS1 proteins differ markedly in secretion efficiency and in their effects on host cells. These findings highlight the value of monitoring WNV NS1 in patients, in contrast to USUV. The detection of WNV NS1 in patient serum and cerebrospinal fluid, together with its early secretion and established role in pathogenesis, underscores its potential as a valuable immunological target. Beyond its use as a diagnostic marker, WNV NS1 may also serve as an indicator of pathogenic processes, particularly those involving disruption of BBB integrity, as previously reported for other orthoflaviviruses. Overall, these insights advance our understanding of NS1-driven mechanisms in orthoflavivirus pathogenesis and support the development of NS1-focused strategies for diagnosis and prevention.

## Supplementary Material

Fig S5.tif

Fig S2.tif

Fig S4.tif

Fig S1.TIF

Fig S3.tif

Supp Table 1 2 3.xlsx

## Data Availability

All data generated or analyzed during this study are included in this published article and its supplementary information files. Additional data are available from the corresponding author upon reasonable request.

## References

[1] Pfeffer M, Dobler G. Emergence of zoonotic arboviruses by animal trade and migration. Parasit Vectors. 2010;3(1):35. doi:10.1186/1756-3305-3-35 20377873 PMC2868497

[2] Simonin Y. Circulation of West Nile virus and Usutu virus in Europe: overview and challenges. Viruses. 2024;16(4):599. doi:10.3390/v16040599 38675940 PMC11055060

[3] Laverdeur J, Amory H, Beckers P, et al. West Nile and Usutu viruses: current spreading and future threats in a warming northern Europe. Front Virol. 2025;5:1544884. doi:10.3389/fviro.2025.1544884

[4] Zannoli S, Sambri V. West Nile virus and Usutu virus co-circulation in Europe: epidemiology and implications. Microorganisms. 2019;7(7):184. doi:10.3390/microorganisms7070184 31248051 PMC6680635

[5] Zerbato V, Rossi B, Di Bella S, et al. West Nile virus: epidemiology, prevention, clinical features, diagnosis, treatment, and open research questions. Ann Med. 2026;58(1):2615482. doi:10.1080/07853890.2026.2615482 41546488 PMC12818317

[6] Cadar D, Simonin Y. Human Usutu virus infections in Europe: a new risk on horizon? Viruses. 2022;15(1):77. doi:10.3390/v15010077 36680117 PMC9866956

[7] Marino A, Vitale E, Maniaci A, et al. West Nile virus: insights into microbiology, epidemiology, and clinical burden. Acta Microbiol Hell. 2025;70(4):44. doi:10.3390/amh70040044

[8] Fall G, Di Paola N, Faye M, et al. Biological and phylogenetic characteristics of West African lineages of West Nile virus. PLoS Negl Trop Dis. 2017;11(11):e0006078. doi:10.1371/journal.pntd.0006078 29117195 PMC5695850

[9] Heus P, Kolodziejek J, Camp JV, et al. Emergence of West Nile virus lineage 2 in Europe: characteristics of the first seven cases of West Nile neuroinvasive disease in horses in Austria. Transbound Emerg Dis. 2020;67(3):1189–1197. doi:10.1111/tbed.13452 31840920 PMC7317211

[10] Hernández-Triana LM, Jeffries CL, Mansfield KL, et al. Emergence of West Nile virus lineage 2 in Europe: a review on the introduction and spread of a mosquito-borne disease. Front Public Health. 2014;2:271. doi:10.3389/fpubh.2014.00271 25538937 PMC4258884

[11] Barzon L, Papa A, Lavezzo E, et al. Phylogenetic characterization of Central/Southern European lineage 2 West Nile virus: analysis of human outbreaks in Italy and Greece, 2013–2014. Clin Microbiol Infect. 2015;21(12):1122.e1–10. doi:10.1016/j.cmi.2015.07.018 26235197

[12] Percivalle E, Cassaniti I, Sarasini A, et al. West Nile or usutu virus? A three-year follow-up of humoral and cellular response in a group of asymptomatic blood donors. Viruses. 2020;12(2):157. doi:10.3390/v12020157 PMC7077259 32013152

[13] Pecorari M, Longo G, Gennari W, et al. First human case of Usutu virus neuroinvasive infection, Italy, August–September 2009. Euro Surveill. 2009;14(50):19446. doi:10.2807/ese.14.50.19446-en 20070936

[14] Simonin Y, Sillam O, Carles MJ, et al. Human Usutu virus infection with atypical neurologic presentation, Montpellier, France, 2016. Emerg Infect Dis. 2018;24(5):875–878. doi:10.3201/eid2405.171122 29664365 PMC5938765

[15] Cadar D, Lühken R, Jeugd Hvd, et al. Widespread activity of multiple lineages of Usutu virus, Western Europe, 2016. Euro Surveill. 2017;22(4):30452. doi:10.2807/1560-7917.ES.2017.22.4.30452 28181903 PMC5388094

[16] Vilibic-Cavlek T, Petrovic T, Savic V, et al. Epidemiology of Usutu virus: the European scenario. Pathogens. 2020;9(9):699. doi:10.3390/pathogens9090699 32858963 PMC7560012

[17] Clé M, Constant O, Barthelemy J, et al. Differential neurovirulence of Usutu virus lineages in mice and neuronal cells. J Neuroinflammation. 2021;18(1):11. doi:10.1186/s12974-020-02060-4 33407600 PMC7789689

[18] Prat M, Jeanneau M, Rakotoarivony I, et al. Virulence and transmission vary between Usutu virus lineages in Culex pipiens. PLoS Negl Trop Dis. 2024;18(6):e0012295. doi:10.1371/journal.pntd.0012295 38935783 PMC11236178

[19] Constant O, Maarifi G, Barthelemy J, et al. Differential effects of Usutu and West Nile viruses on neuroinflammation, immune cell recruitment and blood–brain barrier integrity. Emerg Microbes Infect. 2023;12(1):2156815. doi:10.1080/22221751.2022.2156815 36495563 PMC9815434

[20] Clé M, Barthelemy J, Desmetz C, et al. Study of Usutu virus neuropathogenicity in mice and human cellular models. PLoS Negl Trop Dis. 2020;14(4):e0008223. doi:10.1371/journal.pntd.0008223 32324736 PMC7179837

[21] Revel J, Desmetz C, Simonin Y. The viral protein NS1: a major player in the pathogenesis of orthoflaviviruses. Virologie. 2024;28(3):187–197. doi:10.1684/vir.2024.1050 38970340

[22] Puerta-Guardo H, Glasner DR, Espinosa DA, et al. Flavivirus NS1 triggers tissue-specific vascular endothelial dysfunction reflecting disease tropism. Cell Rep. 2019;26(6):1598–1613. doi:10.1016/j.celrep.2019.01.036 30726741 PMC6934102

[23] Puerta-Guardo H, Glasner DR, Harris E. Dengue virus NS1 disrupts the endothelial glycocalyx, leading to hyperpermeability. PLoS Pathog. 2016;12(7):e1005738. doi:10.1371/journal.ppat.1005738 27416066 PMC4944995

[24] Puerta-Guardo H, Biering SB, Sousa Fd, et al. Flavivirus NS1 triggers tissue-specific disassembly of intercellular junctions leading to barrier dysfunction and vascular leak in a GSK-3β-dependent manner. Pathogens. 2022;11(6):615. doi:10.3390/pathogens11060615 35745469 PMC9228372

[25] Chen HR, Chuang YC, Lin YS, et al. Dengue virus nonstructural protein 1 induces vascular leakage through macrophage migration inhibitory factor and autophagy. PLoS Negl Trop Dis. 2016;10(7):e0004828. doi:10.1371/journal.pntd.0004828 27409803 PMC4943727

[26] Pan P, Li G, Shen M, et al. DENV NS1 and MMP-9 cooperate to induce vascular leakage by altering endothelial cell adhesion and tight junction. PLoS Pathog. 2021;17(7):e1008603. doi:10.1371/journal.ppat.1008603 34310658 PMC8341711

[27] Glasner DR, Ratnasiri K, Puerta-Guardo H, et al. Dengue virus NS1 cytokine-independent vascular leak is dependent on endothelial glycocalyx components. PLoS Pathog. 2017;13(11):e1006673. doi:10.1371/journal.ppat.1006673 29121099 PMC5679539

[28] Beatty PR, Puerta-Guardo H, Killingbeck SS, et al. Dengue virus NS1 triggers endothelial permeability and vascular leak that is prevented by NS1 vaccination. Sci Transl Med. 2015;7(304):304ra141. doi:10.1126/scitranslmed.aaa3787 26355030

[29] Modhiran N, Watterson D, Muller DA, et al. Dengue virus NS1 protein activates cells via toll-like receptor 4 and disrupts endothelial cell monolayer integrity. Sci Transl Med. 2015;7(304):304ra142. doi:10.1126/scitranslmed.aaa3863 26355031

[30] Safadi DE, Lebeau G, Lagrave A, et al. Extracellular vesicles are conveyors of the NS1 toxin during dengue virus and Zika virus infection. Viruses. 2023;15(2):364. doi:10.3390/v15020364 36851578 PMC9965858

[31] Nikolay B, Weidmann M, Dupressoir A, et al. Development of a Usutu virus specific real-time reverse transcription PCR assay based on sequenced strains from Africa and Europe. J Virol Methods. 2014;197:51–54. doi:10.1016/j.jviromet.2013.08.039 24036076

[32] Cecchelli R, Aday S, Sevin E, et al. A stable and reproducible human blood-brain barrier model derived from hematopoietic stem cells. PLoS One. 2014;9(6):e99733. doi:10.1371/journal.pone.0099733 24936790 PMC4061029

[33] Benzarti E, Sarlet M, Franssen M, et al. New insights into the susceptibility of immunocompetent mice to Usutu virus. Viruses. 2020;12(2):189. doi:10.3390/v12020189 32046265 PMC7077335

[34] Samuel MA, Diamond MS. Alpha/beta interferon protects against lethal West Nile virus infection by restricting cellular tropism and enhancing neuronal survival. J Virol. 2005;79(21):13350–13361. doi:10.1128/jvi.79.21.13350-13361.2005 16227257 PMC1262587

[35] Gutsche I, Coulibaly F, Voss JE, et al. Secreted dengue virus nonstructural protein NS1 is an atypical barrel-shaped high-density lipoprotein. PNAS. 2011;108(19):8003–8008. doi:10.1073/pnas.1017338108 21518917 PMC3093454

[36] Somnuke P, Hauhart RE, Atkinson JP, et al. N-linked glycosylation of dengue virus NS1 protein modulates secretion, cell-surface expression, hexamer stability, and interactions with human complement. Virology. 2011;413(2):253–264. doi:10.1016/j.virol.2011.02.022 21429549 PMC3089955

[37] Tan BEK, Beard MR, Eyre NS. Identification of key residues in dengue virus NS1 protein that are essential for its secretion. Viruses. 2023;15(5):1102. doi:10.3390/v15051102 37243188 PMC10221731

[38] Huang J, Wang W, Yu T, et al. NS1: a promising novel target antigen with strong immunogenicity and protective efficacy for avian flavivirus vaccine development. Poult Sci. 2024;103(4):103469. doi:10.1016/j.psj.2024.103469 38335667 PMC10864804

[39] Modhiran N, Song H, Liu L, et al. A broadly protective antibody that targets the flavivirus NS1 protein. Science. 2021;371(6525):190–194. doi:10.1126/science.abb9425 33414219

[40] Hui L, Nie Y, Li S, et al. Matrix metalloproteinase 9 facilitates Zika virus invasion of the testis by modulating the integrity of the blood-testis barrier. PLoS Pathog. 2020;16(4):e1008509. doi:10.1371/journal.ppat.1008509 32302362 PMC7190178

[41] Macdonald J, Tonry J, Hall RA, et al. NS1 protein secretion during the acute phase of West Nile virus infection. J Virol. 2005;79(22):13924–13933. doi:10.1128/jvi.79.22.13924-13933.2005 16254328 PMC1280181

[42] Ding XX, Li XF, Deng YQ, et al. Development of a double antibody sandwich ELISA for West Nile virus detection using monoclonal antibodies against non-structural protein 1. PLoS One. 2014;9(10):e108623. doi:10.1371/journal.pone.0108623 25303282 PMC4193763

[43] Chung KM, Diamond MS. Defining the levels of secreted non-structural protein NS1 after West Nile virus infection in cell culture and mice. J Med Virol. 2008;80(3):547–556. doi:10.1002/jmv.21091 18205232 PMC2696118

[44] Rebollo B, Llorente F, Pérez-Ramírez E, et al. Absence of protection from West Nile virus disease and adverse effects in red legged partridges after non-structural NS1 protein administration. Comp Immunol Microbiol Infect Dis. 2018;56:30–33. doi:10.1016/j.cimid.2017.12.006 29406280

[45] Ruggiano A, Foresti O, Carvalho P. ER-associated degradation: protein quality control and beyond. J Cell Biol. 2014;204(6):869–879. doi:10.1083/jcb.201312042 24637321 PMC3998802

[46] Daniel RM, Cowan DA, Morgan HW, et al. A correlation between protein thermostability and resistance to proteolysis. Biochem J. 1982;207(3):641–644. doi:10.1042/bj2070641 6819862 PMC1153914

[47] Lo NTN, Roodsari SZ, Tin NL, et al. Molecular determinants of tissue specificity of flavivirus nonstructural protein 1 interaction with endothelial cells. J Virol. 2022;96(19):e0066122. doi:10.1128/jvi.00661-22 36106873 PMC9555157

[48] Akey DL, Brown WC, Dutta S, et al. Flavivirus NS1 structures reveal surfaces for associations with membranes and the immune system. Science. 2014;343(6173):881–885. doi:10.1126/science.1247749 24505133 PMC4263348

[49] Wessel AW, Dowd KA, Biering SB, et al. Levels of circulating NS1 impact West Nile virus spread to the brain. J Virol. 2021;95(20):e0084421. doi:10.1128/jvi.00844-21 34346770 PMC8475509

[50] Roe K, Kumar M, Lum S, et al. West Nile virus-induced disruption of the blood-brain barrier in mice is characterized by the degradation of the junctional complex proteins and increase in multiple matrix metalloproteinases. J Gen Virol. 2012;93(6):1193–1203. doi:10.1099/vir.0.040899-0 22398316 PMC3755517

[51] Verma S, Kumar M, Gurjav U, et al. Reversal of West Nile virus-induced blood-brain barrier disruption and tight junction proteins degradation by matrix metalloproteinases inhibitor. Virology. 2010;397(1):130–138. doi:10.1016/j.virol.2009.10.036 19922973 PMC3102050

[52] Velandia-Romero ML, Calderón-Peláez MA, Castellanos JE. In vitro infection with dengue virus induces changes in the structure and function of the mouse brain endothelium. PLoS One. 2016;11(6):e0157786. doi:10.1371/journal.pone.0157786 27336851 PMC4919088

[53] Puerta-Guardo H, Biering SB, Castillo-Rojas B, et al. Flavivirus NS1-triggered endothelial dysfunction promotes virus dissemination. bioRxiv. 2024. doi:10.1101/2024.11.29.625931 PMC12779140 41474801

[54] Khlusevich Y, Kravchuk B, Kechin A, et al. TBEV NS1 induces tissue-specific microvascular endothelial cell permeability by activating the TNF-α signaling pathway. Int J Mol Sci. 2025;26(11):5311. doi:10.3390/ijms26115311 40508120 PMC12154905

[55] Llorente F, Gutiérrez-López R, Pérez-Ramirez E, et al. Experimental infections in red-legged partridges reveal differences in host competence between West Nile and Usutu virus strains from Southern Spain. Front Cell Infect Microbiol. 2023;13:1163467. doi:10.3389/fcimb.2023.1163467 37396301 PMC10308050

[56] Olofsson J, Tolf C, Lindqvist R, et al. Evidence of exposure to West Nile virus and Usutu virus in migratory birds in Sweden. IJID One Health. 2024;5:100039. doi:10.1016/j.ijidoh.2024.100039

