# Supplementary figures and images for: Differential properties of NS1 glycoproteins in West Nile and Usutu viruses
Source: Emerg Microbes Infect. 2026 Apr 29;15(1):2667565. doi: 10.1080/22221751.2026.2667565 (PMC13224725; doi:10.1080/22221751.2026.2667565)

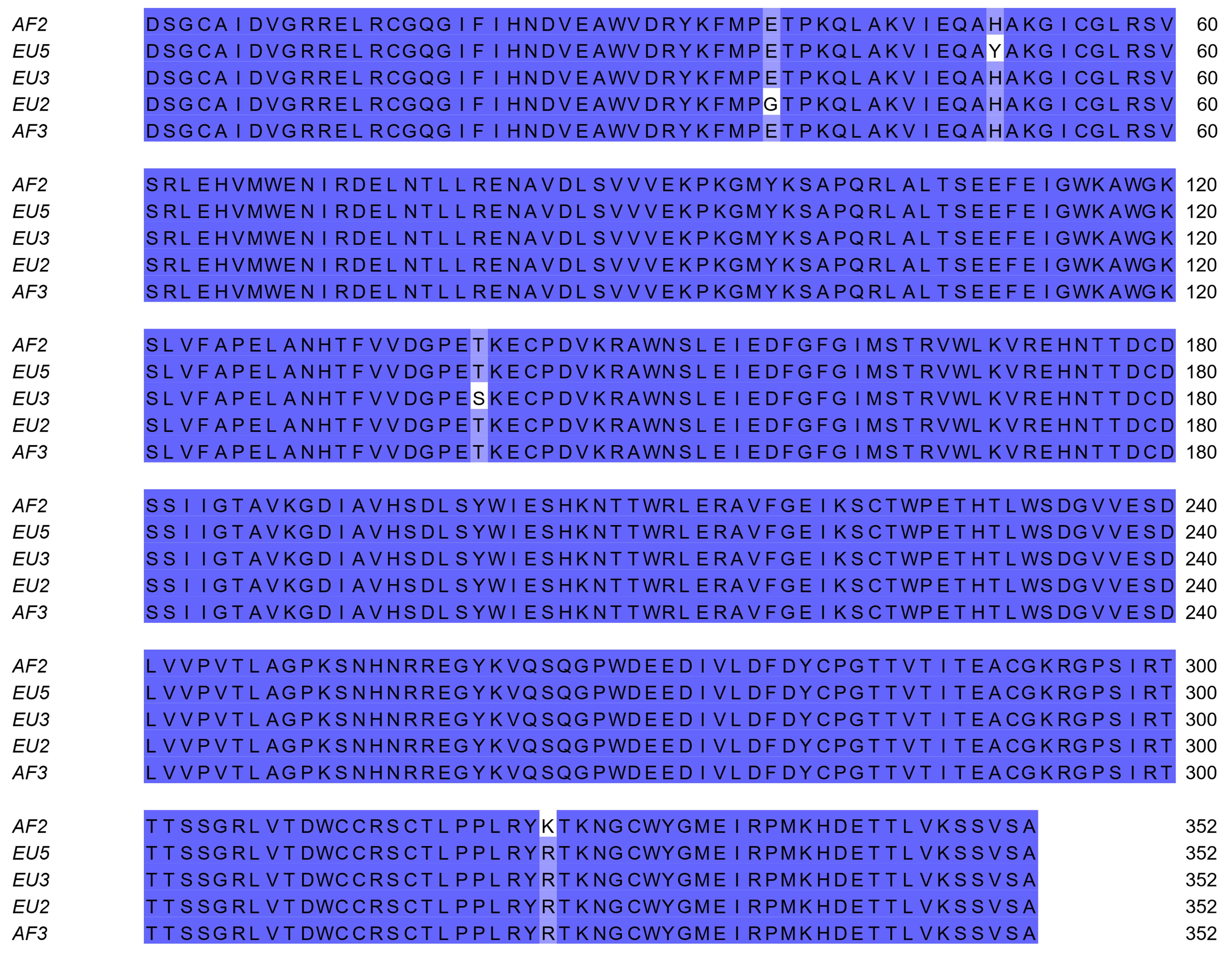

Supplement: Fig S5.tif [file TEMI_A_2667565_SM3025.tif]

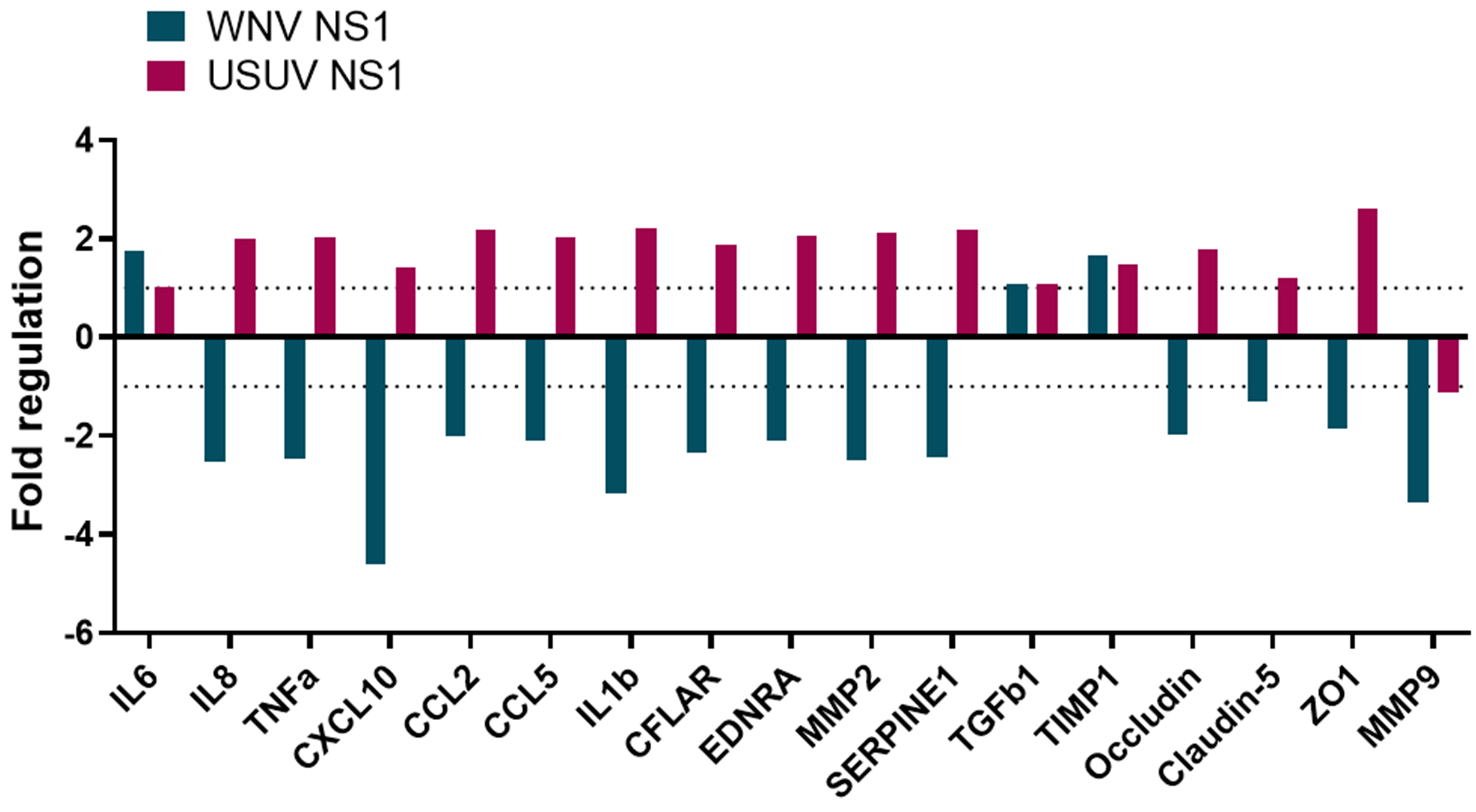

Supplement: Fig S2.tif [file TEMI_A_2667565_SM3024.tif]

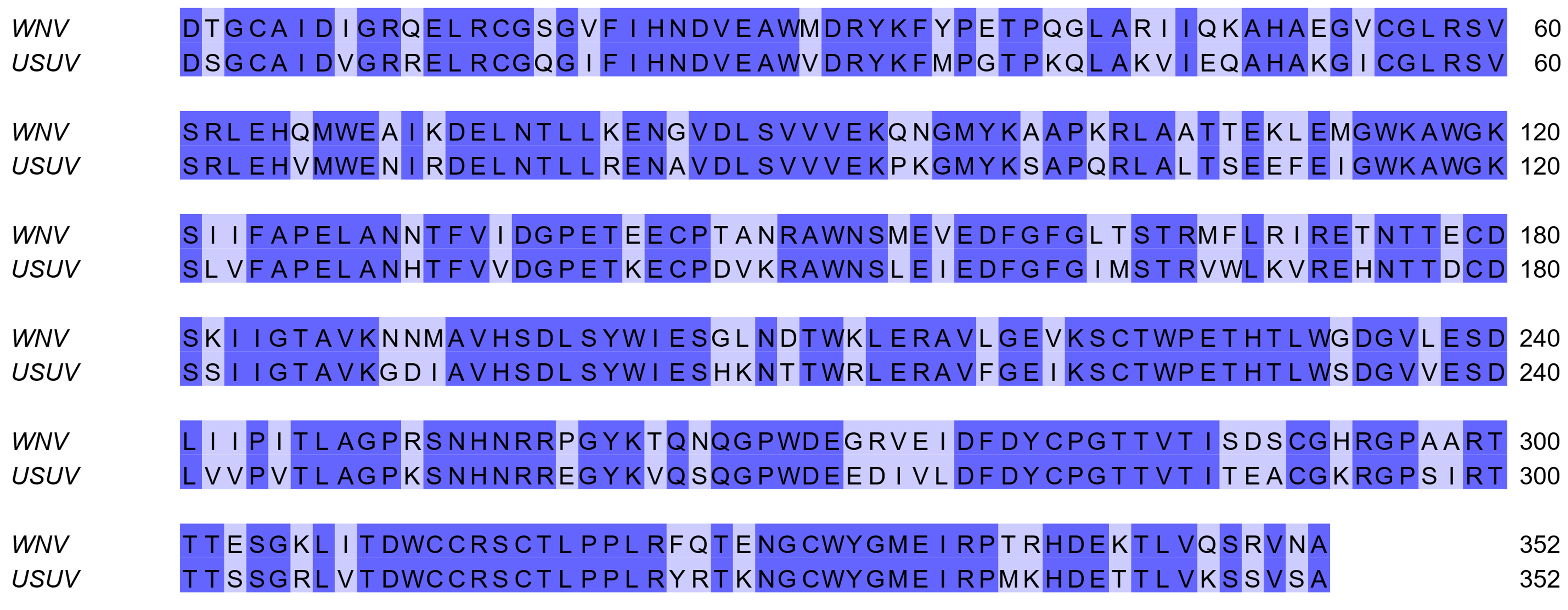

Supplement: Fig S4.tif [file TEMI_A_2667565_SM3023.tif]

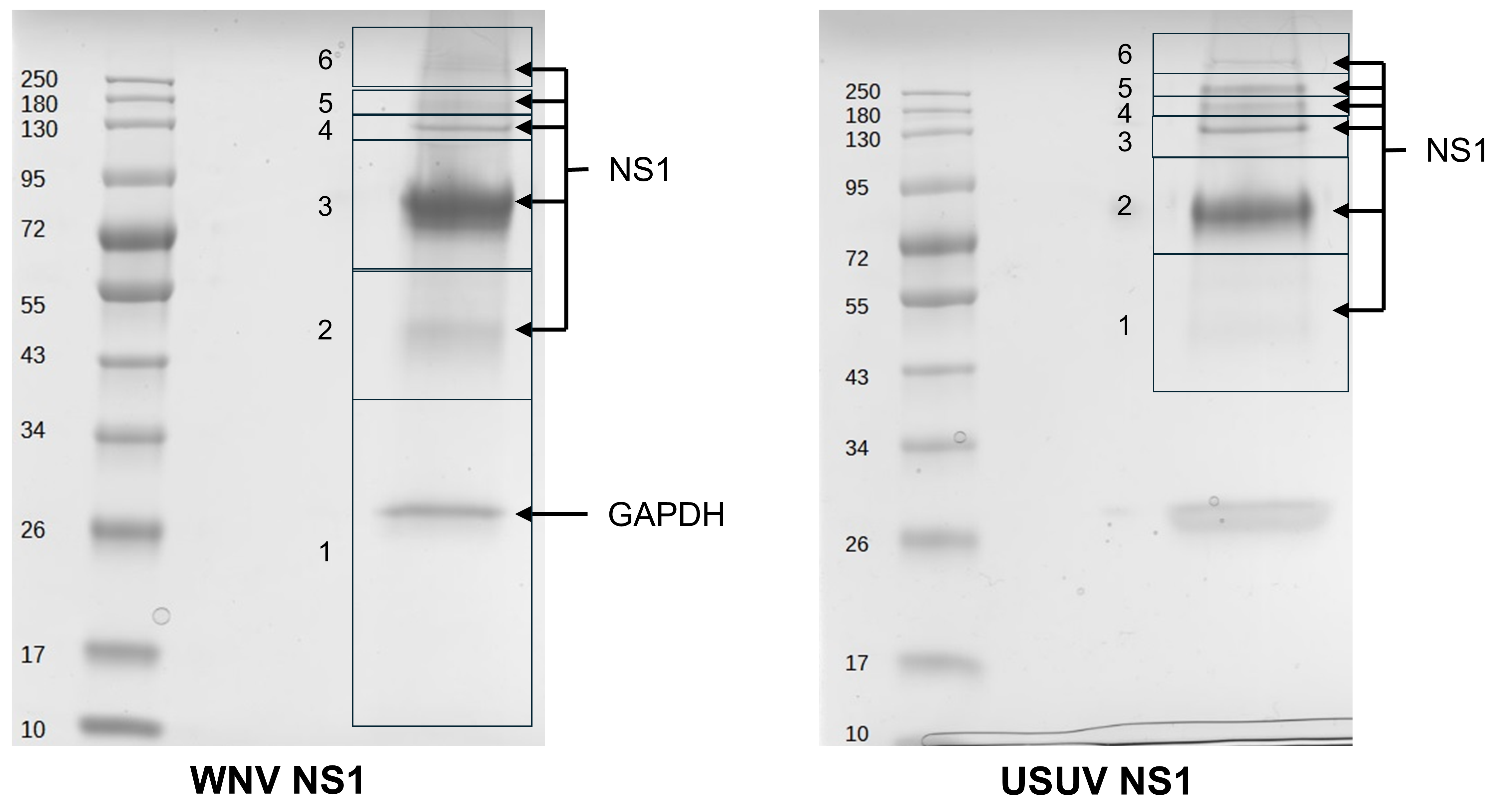

Supplement: Fig S1.TIF [file TEMI_A_2667565_SM3022.tif]

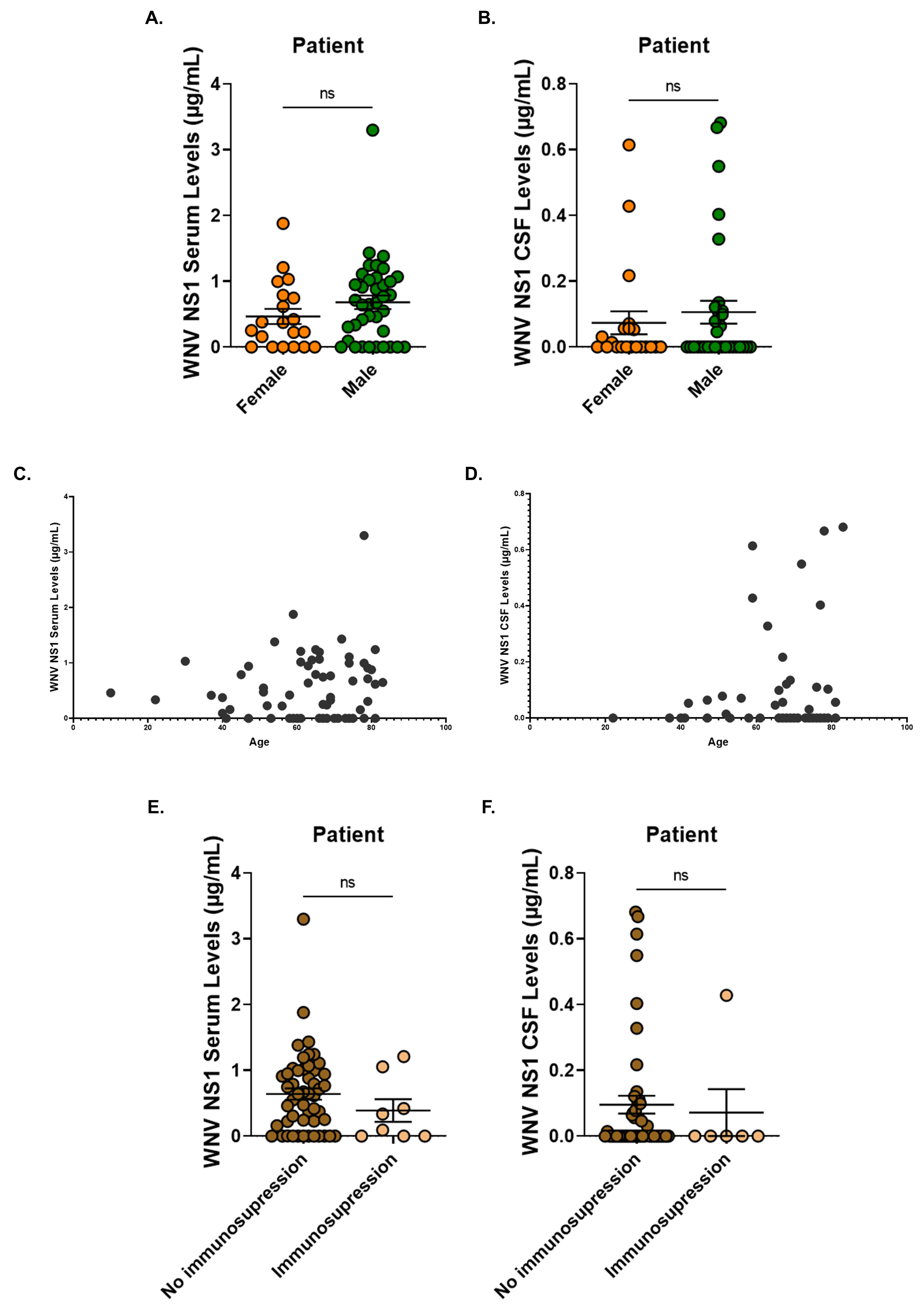

Supplement: Fig S3.tif [file TEMI_A_2667565_SM3021.tif]
